# The impact of surgical simulation on patient outcomes: a systematic review and meta-analysis

**DOI:** 10.1007/s10143-020-01314-2

**Published:** 2020-05-13

**Authors:** Trym R. Meling, Torstein R. Meling

**Affiliations:** 1 Faculty of Medicine, University of Oslo, Oslo, Norway; 2 Department of Clinical Neurosciences, Division of Neurosurgery, Geneva University Hospitals, Rue Gabriel-Perret-Gentil 5, 1205 Geneva, Switzerland; 3 Faculty of Medicine, University of Geneva, Geneva, Switzerland

**Keywords:** Education, Meta-analysis, Neurosurgery, Patient outcome, Simulation, Surgery, Systematic review

## Abstract

The use of simulation in surgical training is ever growing. Evidence suggests such training may have beneficial clinically relevant effects. The objective of this research is to investigate the effects of surgical simulation training on clinically relevant patient outcomes by evaluating randomized controlled trials (RCT). PubMed was searched using PRISMA guidelines: “surgery” [All Fields] AND “simulation” [All Fields] AND “patient outcome” [All Fields]. Of 119 papers identified, 100 were excluded for various reasons. Meta-analyses were conducted using the inverse-variance random-effects method. Nineteen papers were reviewed using the CASP RCT Checklist. Sixteen studies looked at surgical training, two studies assessed patient-specific simulator practice, and one paper focused on warming-up on a simulator before performing surgery. Median study population size was 22 (range 3–73). Most articles reported outcome measures such as post-intervention Global Rating Scale (GRS) score and/or operative time. On average, the intervention group scored 0.42 (95% confidence interval 0.12 to 0.71, *P* = 0.005) points higher on a standardized GRS scale of 1–10. On average, the intervention group was 44% (1% to 87%, *P* = 0.04) faster than the control group. Four papers assessed the impact of simulation training on patient outcomes, with only one finding a significant effect. We found a significant effect of simulation training on operative performance as assessed by GRS, albeit a small one, as well as a significant reduction to operative time. However, there is to date scant evidence from RCTs to suggest a significant effect of surgical simulation training on patient outcomes.

**Electronic supplementary material:**

The online version of this article (10.1007/s10143-020-01314-2) contains supplementary material, which is available to authorized users.

## Introduction

Surgical training has traditionally been based on an apprenticeship model [1, 2], to which there are substantial benefits. Prime among them is authenticity; one is exposed to all the concerns, pressures, and emotions that surgical practice entails. Furthermore, expert surgeons may use the operating room (OR) as a classroom [2], providing apprentices with instruction and feedback in a live setting, thus enhancing the role of the expert as a scaffold for the trainee [3]. However, this model has some important drawbacks. With the modern-day focus on ethical treatment standards [4], there is concern about “learning on the job” and its implications for patient safety [5, 6]. In the current climate of long waiting lists and schedules swamped by consultations and paperwork, the ethical and monetary costs to spending the time of expert surgeons necessary to train novices have never been higher [7, 8]. Furthermore, legislation has been introduced in Europe and the USA to limit the work hours of surgical residents [9, 10]. The European Working Time Directive was designed to prevent excessively long work hours [11], and thus, by extension, better and safer patient care. However, concerns have been raised about unintended consequences [9, 12, 13], such as dilution to the quantity and quality of training opportunities [12].

This has prompted the exploration of alternatives, such as simulation tools [14], that range from the basic table-top box trainer, to porcine cadavers, and state-of-the-art virtual reality (VR) simulators [15–26]. Such tools may potentiate the amount of repetitions necessary to gain automaticity in surgical techniques [2, 27], provide a risk-free environment in which no patients health is in jeopardy [28, 29], and allow for practice of procedures that are performed infrequently. Simulation centers can be accessible around the clock, allowing for considerable flexibility in scheduling. Another interesting prospect is the possibility of establishing a baseline of competence on simulators that must be shown by residents before being allowed to advance, giving trainees and their supervisors confidence in ability and ensuring a certain performance standard in the OR [30].

When investigating novel modes of teaching, it is important to establish benefits, as well as limitations [14]. It has been shown that practice of skills in a simulated setting leads to improvement of those skills when tested in that same environment [31–33], but this outcome appears self-evident. To the authors of this paper, a natural area of inquiry is transferability to the clinical setting. For example, maneuvering a colonoscope while wearing a VR headset is a different milieu to performing the examination on an uneasy patient or that the concerns when operating on a human patient differ from those when manipulating the tissues of an anesthetized pig. So, to what extent does surgical simulation training translate to performance in the OR and more importantly, what are its effects on clinically relevant patient outcomes? To answer this query, a systematic review of the relevant literature on surgical simulation skills training was conducted.

## Methods

Evaluation of studies on simulation training was performed according to the Preferred Reporting Items for Systematic Reviews and Meta-Analyses (PRISMA) statement, including the methods of publication search, eligibility, data collection, extraction, and analysis, as well as preparation of the systematic review report [34, 35]. Patients and the public were not involved in this research.

**Fig. 1:**
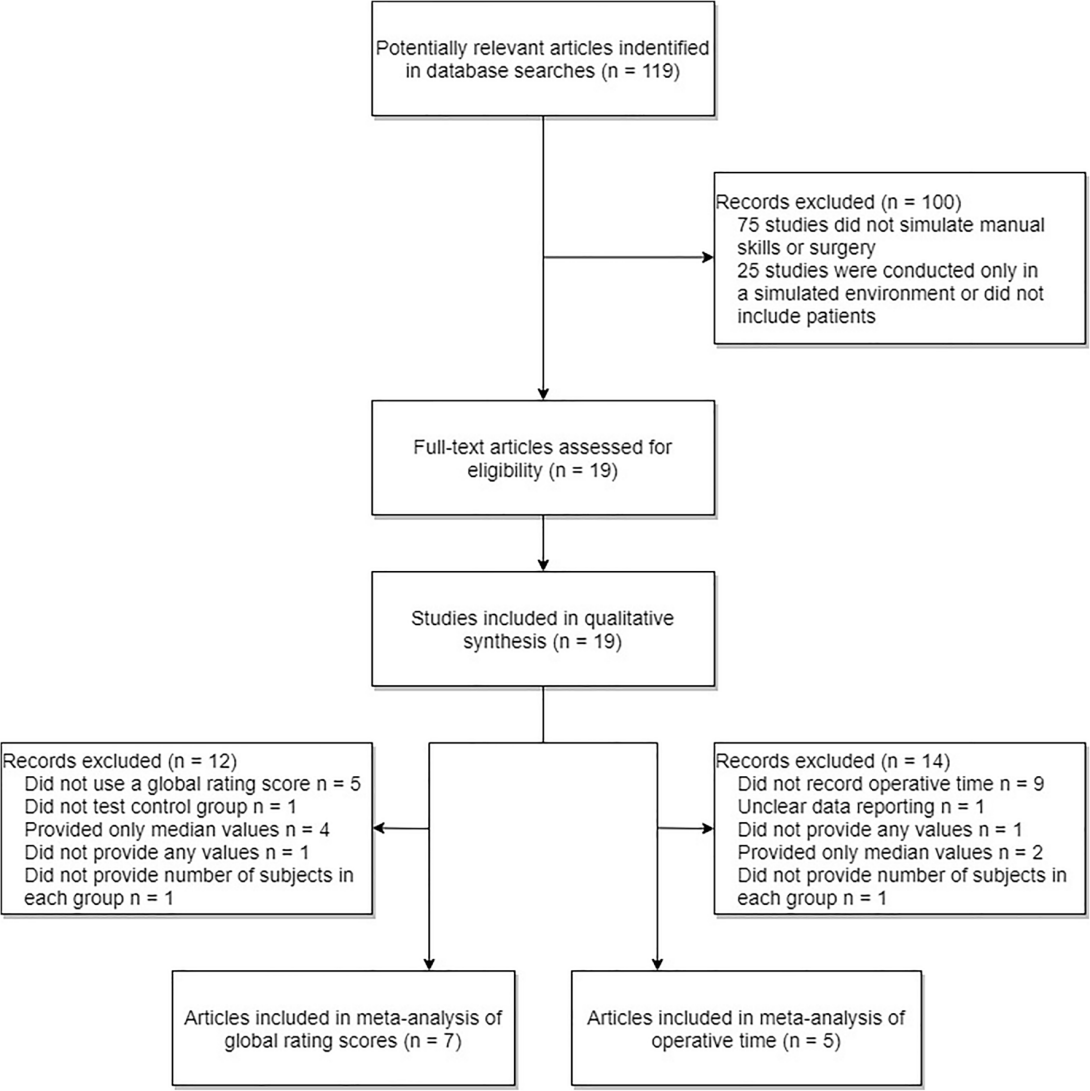
PRISMA flow diagram visualizing the literature search. A total of 119 papers were identified in our literature search. One hundred of these were excluded; the remaining 19 papers were assessed for eligibility and subsequently included in qualitative analysis. Of these, seven were eligible for inclusion in our meta-analysis of global rating scores and six in our analysis of operative time

A study protocol was created, available as a supplemental file (Supplemental Digital Content File 1). A search of PubMed databases using the following criteria “surgery” [All Fields] AND “simulation” [All Fields] AND “patient outcome” [All Fields] was performed on January 7, 2019. The following search filters were applied: human species and randomized controlled trial article type. A PRISMA flow diagram was created in order to visualize this process (Fig. 1) [36]. The literature search produced 119 papers, the abstracts of which were reviewed by two raters to determine eligibility as per convention to determine relevancy (Supplemental Digital Content File 2). Studies were excluded if they did not involve surgery (e.g., vaginal child delivery) or if the simulation described lacked involvement of manual skills (e.g., three-dimensional reconstruction of a cyst to plan surgery). This resulted in the exclusion of 75 papers. Studies were further excluded if no patient treatments were conducted or if all measurements were recorded in a simulated setting. This led to the removal of another 25 papers. The full text of the remaining 19 papers were reviewed using the Critical Appraisal Skills Programme (CASP)–Randomized Controlled Trial (RCT) Checklist as a guide (Supplemental Digital Content File 3) [37].

Information sought in each paper was the following: study population enrolled, study population in the final analysis, training level of study population, type of procedure studied, whether or not an OR baseline was established, use of intraoperative rating scale, recording of operative time, type of simulation used, time allotted to simulation training, type of training control group received, if the intervention group was significantly better than the control group after intervention, clinically relevant effects of training, if intervention group improved from its baseline following intervention, and effect of intervention on patient outcomes.

Study quality was assessed by evaluating the mode of randomization used, if the trial was stopped early, if patient assignment was truly randomized (that is to say, assignment was done by a random number generator or similar process, not merely dependent on the surgical staff rotation), blinding of patients and data collectors, accounting of subjects at trial conclusion, evidence of selective reporting, and similarity of groups at the start of the trial.

Patient outcomes were defined as data or measurements of patients made after surgical intervention; in-hospital and 30-day mortality, as well as postoperative complications, for example. In contrast, operative time and subjective assessments of perioperative errors were not considered patient outcomes.

Study data measured in a clinical setting (i.e., on real patients, not in a simulated environment) were extracted by the first author and added to a data sheet (Supplemental Digital Content File 4). One study was reported in two separate papers, one of which was identified in our literature search (Desender et al. [38]) and included in the final review. Relevant data and results were extracted from the other paper [39] and are considered one under the guise of the former, as they share the same first author.

The majority of the articles reported outcome measures such as Global Rating Scale (GRS) score and operative time. Rating scales are tools for the objective evaluation of an individual’s skill, as assessed by experts or trained personnel. Typically, they consist of seven items, scored on a Likert scale of 1–5 [40]. Many such instruments are in use; they may concern general operative performance or be specific to a certain procedure [41, 42]. There is some heterogeneity in terms of number of items (e.g., 4 or 10) as well as Likert scale range employed by different GRSs. To accommodate this and allow for the combination of data, an arbitrary scale of 1–10 was created by authors in order to standardize values across studies. The GRS used by each study was then mapped to this arbitrary scale and a standardized mean was calculated (Supplemental Digital Content File 5). In cases where a study used multiple rating scales, the most generic one was chosen.

Different surgical procedures require different amounts of time to complete. Thus, to compare operative times, the mean result of the intervention group and that of the control group was divided by the result of the control group. In this way, the control mean becomes 1 for all studies, and the intervention mean is expressed as a fraction of the control, with a value smaller or larger than 1, depending on whether the group was faster or slower (Supplemental Digital Content File 5).

If a study had more than two groups, the control group was compared with the main intervention group. If groups were tested at multiple times, the results of the first test post-intervention were used. If a paper reported 95% confidence intervals (CI), the standard deviation was calculated (Supplemental Digital Content File 5). Papers that reported results only as median values were excluded from synthesis. In cases of incomplete data reporting, attempts were made to calculate necessary values (from given *P* values, for example). If such attempts were unsuccessful, the study was excluded from data synthesis. Lack of significant differences between intervention and control groups was not a cause for exclusion from data synthesis. These actions were observed in accordance with the Cochrane Collaboration’s guidelines on conducting meta-analyses outlined in Part 2, Chapter 9 of the *Cochrane Handbook for Systematic Reviews of Interventions* [43].

Data processing was accomplished with the aid of Wolfram|Alpha [44]. Meta-analyses were performed using the Cochrane Collaboration’s Review Manager software [45]. Standardized means were compared with the inverse-variance random-effects method. The effect size is the standardized mean difference, Hedge’s (adjusted) *g* [43]. Heterogeneity was assessed using the chi^2^ and *I*^2^ tests.

## Results

We assessed and included a total of 19 studies in this review. Sixteen studies looked at surgical training, two studies assessed patient-specific simulator practice prior to the actual procedure, and one paper focused on warming-up on a simulator before performing surgery. The median number of enrolled operators was 22 (range 3–73). Ten of the papers assessed surgical residents, two assessed expert surgeons, and the remainder assessed people from other medical backgrounds (Table 1).

**Table 1:** Key characteristics of the 19 papers included in the study

Authors	No. of participants enrolled*	No. in final analysis**	Training level of study population	Procedure simulated	Type of simulator
Wooster et al. [27]	3	3	Expert surgeons	Endovascular (carotid stenting)	VR
Maertens et al [41]	32	29	Surgical residents	Endovascular (angioplasty)	VR
Zevin et al.^56.^	20	8	Surgical residents	Laparoscopic (bariatric surgery)	Porcine model
Desender et al. [29]	NA	NA	Expert surgeons	Endovascular (EVAR)	VR
Nilsson et al. [17]	36	35	Medical students	Endoscopic (camera navigation)	VR
Waterman et al. [46]	22	22	Surgical residents	Endoscopic (shoulder arthroscopy)	VR
Shore et al. [15]	27	21	Surgical residents	Laparoscopic (salpingectomy)	VR and box trainer
Patel et al. [16]	22	22	Surgical residents	Laparoscopic (salpingectomy)	Porcine model
Dunn et al. [47]	17	17	Surgical residents	Endoscopic (shoulder arthroscopy)	VR
Peltan et al. [29]	73	51	Internal medicine residents	CVC placement (internal jugular vein)	Box trainer
Grover et al. [42]	34	33	Surgical and internal medicine residents	Endoscopy (colonoscopy)	VR
Carlsen et al. [48]	18	16	Surgical residents	Open hernia repair (Lichtenstein)	Porcine model and box trainer
Koch et al. [49]	18	18	Internal medicine residents	Endoscopic (colonoscopy)	VR
Zendejas et al. [14]	50	50	Surgical residents	Laparoscopic (TEP inguinal hernia repair)	Box trainer
Kessler et al. [6]	56	32	Pediatric residents	Infant lumbar puncture	Box trainer
Calatayud et al. [30]	10	8	Surgical residents	Laparoscopic (cholecystectomy)	VR
Haycock et al. [50]	40	36	Medical practitioners	Endoscopic (colonoscopy)	VR
Ahlberg et al. [2]	13	13	Surgical residents	Laparoscopic (cholecystectomy)	VR
Cohen et al. [51]	51	45	Internal medicine fellows	Endoscopic (colonoscopy)	VR

*Study population refers to number of surgeons

**Number of completed VR cases; group i was tested twice in patient after 10, 30, and 50 VR cases completed; group ii was tested twice in patients after 20, 60, and 100 VR cases completed

The procedures studied were endoscopic (*n* = 7), laparoscopic (*n* = 6), endovascular (*n* = 3), central venous catheter (CVC) placement (*n* = 1), open hernia repair (*n* = 1), and lumbar puncture (*n* = 1). Five papers established a baseline in the OR before administering the intervention. VR simulators were most commonly used (*n* = 12) for the training intervention; other simulators included box trainers (*n* = 3), porcine models (*n* = 2), and a combination of these (*n* = 2) (Table 1).

The duration of the simulator training differed across the studies included in this review. In most studies, the intervention group practiced for a certain amount of time; five for 2 h or less, five for more than 2 h. Six studies used a predetermined measure of proficiency (such as completing a given task with no mistakes or in a certain amount of time) to determine when an enrollee was ready for testing. Two studies practiced on simulated patient-specific anatomy prior to the actual surgery, with the control group in one of these rehearsing the patient’s anatomy after performing the real procedure. One paper administered simulation training to both its groups, testing them after different numbers of completed cases on a VR simulator. In most of the studies, the control groups merely continued with conventional residency training (*n* = 12) or received no training at all (*n* = 2). In other cases, surgeons served as their own control (*n* = 1), practiced on a simulator without receiving instruction or feedback (*n* = 1), or practiced on real patients instead of on a simulator (*n* = 1) (Table 2).

**Table 2:** Further characteristics of the 19 papers included in the study

Authors	OR baseline established	Use of intraop. rating scale	Recorded operative time	Patient outcomes recorded	Time spent on simulation training	Control group training	Clinically relevant effect	Effect of training on patient outcome
Wooster et al. [27]	No	No	Yes	No	Pre-op. rehearsal ≤ 24 h	None	No	NA
Maertens et al. [41]	No	Yes	Yes	Yes	Time until proficiency	Continued conventional training	Yes	No
Zevin et al. [52]	No	Yes	No	No	Time until proficiency	Continued conventional training	NA	NA
Desender et al. [29]	No	Yes	Yes	Yes	Pre-operative rehearsal once	Rehearsal after procedure	Yes	No
Nilsson et al. [17]	No	Yes	No	No	2 h	None	No	NA
Waterman et al. [46]	Yes	Yes	Yes	No	1 h	Continued conventional training	Yes	NA
Shore et al. [15]	No	Yes	Yes	No	14 h	Continued conventional training	Yes	NA
Patel et al. [6]	Yes	Yes	No	No	1.5 h	Continued conventional training	NA	NA
Dunn et al. [47]	Yes	Yes	Yes	No	1 h	Continued conventional training	No	NA
Peltan et al. [29]	No	Yes	No	Yes	Time until proficiency	Continued conventional training	Yes	No
Grover et al. [42]	No	Yes	No	No	8 h	Simulation training without feedback	Yes	NA
Carlsen et al. [48]	No	Yes	Yes	No	1 day skills lab course	Continued conventional training	Yes	NA
Koch et al. [49]	Yes	No	No	No	Number of procedures**	Number of procedures**	Yes	NA
Zendejas et al. [14]	Yes	Yes	Yes	Yes	Time until proficiency	Continued conventional training	Yes	Yes
Kessler et al. [6]	No	No	No	No	Time until proficiency	Continued conventional training	Yes	NA
Calatayud et al. [30]	No	Yes	No	No	0.75 h	Surgeons served as own controls	Yes	NA
Haycock et al. [50]	No	Yes	Yes	No	16 h	16 h of practice on patients	No	NA
Ahlberg et al. [2]	No	No	Yes	No	Time until proficiency	Continued conventional training	Yes	NA
Cohen et al. [51]	No	No	No	No	10 h	Continued conventional training	Yes	NA

*Study population refers to number of surgeons

**Number of completed VR cases; group i was tested twice in patient after 10, 30, and 50 VR cases completed; group ii was tested twice in patients after 20, 60, and 100 VR cases completed

**Fig. 2:**
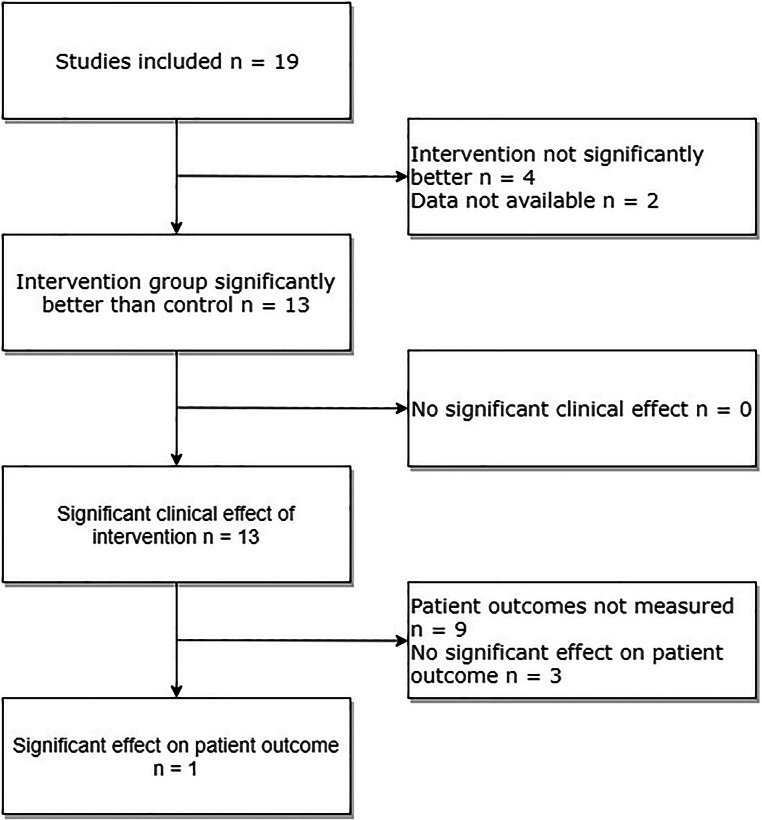
Flow chart visualizing the results of included studies, including the number of papers that demonstrated a clinical effect and/or effect on patient outcomes

In terms of recorded measurements, 14 studies used some form of intraoperative rating scale and 10 studies recorded operative time. Thirteen studies showed a statistically significant difference (of any measure) between control and intervention group at first testing after the intervention; four studies did not find any significant differences, and analysis was not available from two (Table 2). Of the 13 papers that found a significant difference between the intervention and control group, all 13 were judged by the authors of this review to be potentially clinically significant. However, only four of these studies assessed patient outcomes and three found no significant effect of intervention. This was visualized using a flow chart (Fig. 2).

The paper that did find a significant effect of intervention on patient outcome was by Zendejas and colleagues [14], who looked at simulation training of laparoscopic total extra-peritoneal repair of inguinal hernias. Their intervention consisted of an online skills course followed by a multiple-choice questionnaire, after which subjects completed a skills course on a box trainer. Participants were required to complete the assigned task in under 2 min on two consecutive attempts. At post-intervention assessment in the OR, the intervention group was significantly faster, achieved higher GRS scores, and made fewer intraoperative errors. Furthermore, their surgeries resulted in fewer postoperative complications (urinary retention, seroma, hematoma, or wound infection) and overnight hospital stays. Hernia recurrence and groin pain at 3-month follow-up were similar between the control and intervention groups.

Risk of bias and study quality were also assessed, the main results of which are presented in Table 3. Sixteen studies reported how their trial was randomized. One trial was stopped early. Only two papers used a random number generator (or similar tool) to assign patients to study participants. Only two of the papers reviewed reported adequate blinding of patients to the training status of the subject performing their surgery. In 15 studies, all data collectors were blinded to the group subjects belonged to. Only one study did not adequately account for all enrolled subjects at the conclusion of the trial. Seventeen studies reported data on the similarity of its groups at the start of the trial.

**Table 3:** Results of our investigation into the quality and risk of bias of the 19 included studies

Authors	Mode of randomization	RCT stopped early	Patient assignment truly randomized	Patients blinded	Data collectors blinded	Subjects accounted for at trial conclusion	Selective reporting	Groups similar at start of trial?
Wooster et al. [27]	By computer	Yes	Yes	Yes	Yes	Yes	No	NA
Maertens et al. [41]	Sealed envelope	No	No	NA	Yes and no*	Yes	No	Yes, with respect to sex, post-grad year, and number of endovascular cases assisted
Zevin et al. [52]	Sealed envelope	No	No	NA	Yes	Yes	No	Yes, with respect to a host of variables, but intervention group had significantly fewer basic bariatric surgeries performed as the primary surgeon and bariatric rotations participated in
Desender et al. [29]	Sealed envelope	No	Yes	Yes	Yes	NA	No	NA
Nilsson et al. [17]	Sealed envelope	No	No	NA	NA	Yes	No	Yes, with respect to age and experience with laparoscopic training and surgery, but not sex
Waterman et al. [46]	NA	No	No	NA	Yes	Yes	No	Yes, with respect to age, sex, post-grad year, and arthroscopies performed pre and post-intervention
Shore et al. [15]	By computer	No	No	NA	Yes	Yes	No	Yes, with respect to a host of variables (surgical experience, VR experience, musical instrument experience, etc.)
Patel et al. [16]	By computer	No	No	NA	Yes	Yes	Yes****	Groups stratified by pre-intervention human salpingectomy OSAT score, post-grad year was similar
Dunn et al. [47]	NA	No	No	NA	Yes	Yes***	No	Yes with respect to sex, post-grad year, and number of cases performed
Peltan et al. [29]	By computer	No	No	NA	Yes	Yes	No	Yes, with respect to age, sex, training track, and degree
Grover et al. [42]	Sealed envelope	No	No	NA	Yes	Yes	Yes*****	Yes, with respect to age, sex, training program, and number of colonoscopies performed and assisted
Carlsen et al. [48]	Sealed envelope	No	No	NA	Yes	Yes	No	Yes, with respect to age, sex, time in surgical employment, and prior number of performed hernia repairs
Koch et al. [49]	NA	No	No	NA	Yes	Yes	No	Yes, all subjects were at the start of their training in gastroenterology with no previous endoscopic experience
Zendejas et al. [14]	Sealed envelope	No	No	NA	Yes and no**	Yes	No	Yes, baseline TEP repair was similar, groups were similar with respect to a host of other variables (post-grad year, sex, handedness, video game experience, TEP comfort + experience)
Kessler et al. [6]	By computer	No	No	NA	No	Yes	No	Yes, with respect to sex, post-grad year and experience with LP (training, simulator experience, observations, LPs performed)
Calatayud et al. [30]	Sealed envelope	No	No	NA	Yes	Yes	Yes*****	Each surgeon served as their own control
Haycock et al. [50]	By computer	No	No	NA	Yes	Yes	No	Yes, with respect to age, sex, educational direction, sigmoidoscopies and colonoscopies witnessed/assisted/performed
Ahlberg et al. [2]	Sealed envelope	No	No	NA	Yes	Yes	No	Yes, with respect to age, sex, visuospatial assessment, working memory assessment, and laparoscopic assisting experience
Cohen et al. [51]	Random number table	No	No	NA	Yes	Yes	No	Yes, with respect to experience with gastroscopy and flexible sigmoidoscopies

*Blinded supervising surgeon was responsible for Global Rating Scale score and Examiner Checklist score, all other outcomes recorded by non-blinded investigator

**Blinded supervising surgeon was responsible for GOALS score and postoperative complications, all other outcomes recorded by non-blinded investigator group

***Paper specifies total subjects enrolled and analyzed, but not how many subjects were in control and intervention

****Fail to report any between group analysis, only reporting within group

*****Assessments are based on review of video recording of surgical procedures, but the investigators do not mention or conduct analysis of operative time, which should be readily available to them

### Effect on Global Rating Scale

Fourteen papers evaluated its subjects using a GRS. Of these, four studies were excluded due to only reporting median values and one due to not testing its control group outside of a simulated setting. One study only presented statistical analysis without reporting raw data or means; attempts were made to reconstruct these values without success. One study presented the total number of subjects enrolled, without mentioning the sizes of its control and intervention group. Attempts were made to calculate this given reported *P* values, but to no avail. As such, only a total of seven studies had the necessary data quality to be included in our analysis.

**Fig. 3:**
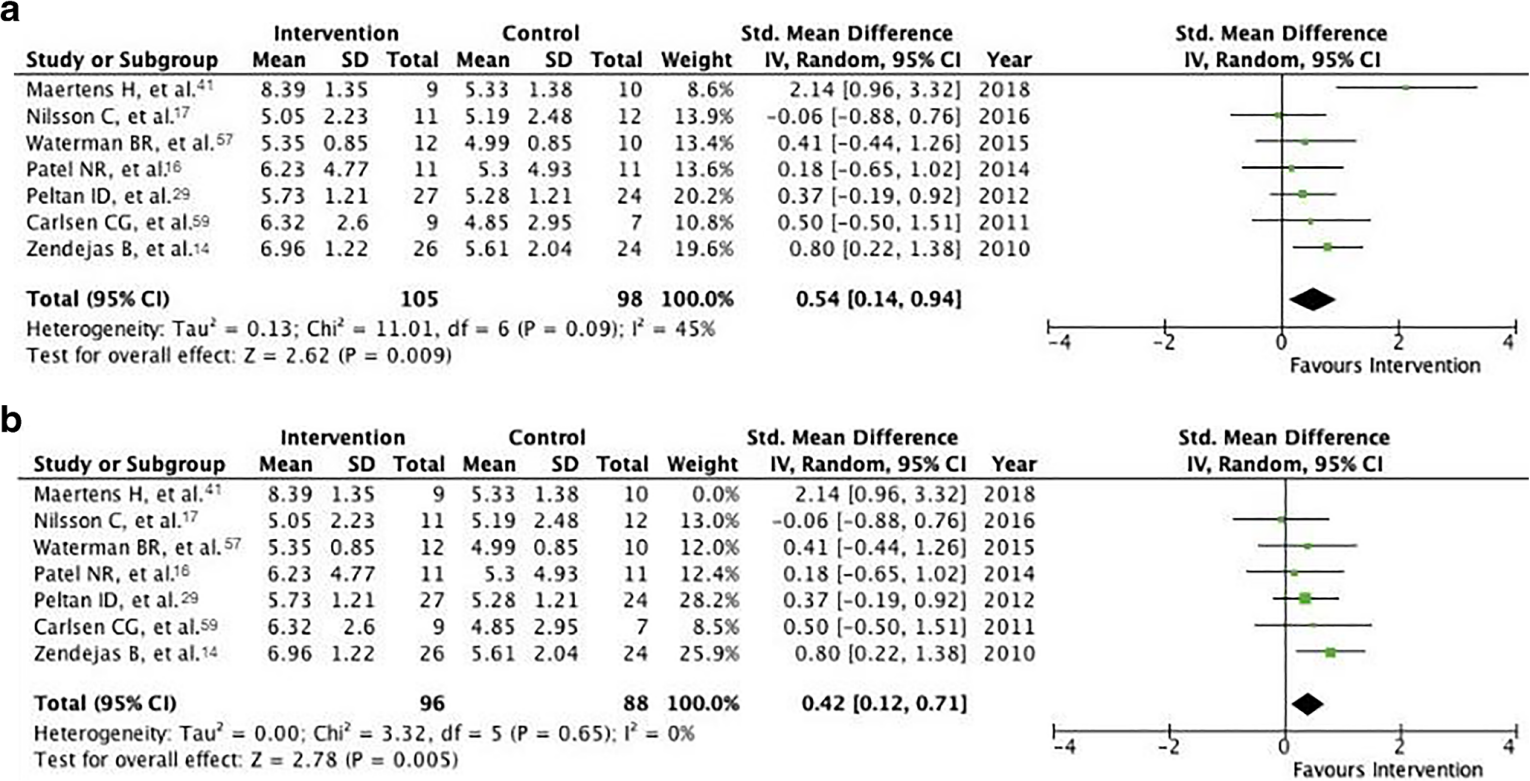
**a** Forest plot of the standardized mean difference of GRS scores between control and intervention groups, including tests for heterogeneity. **b** Forest plot of the standardized mean difference of GRS scores between control and intervention groups with outlier removed, including tests for heterogeneity

The standardized mean difference was 0.54 (95% confidence interval 0.14 to 0.94, *P* = 0.009) (Fig. 3a). Thus, in these seven papers, the intervention group scored on average 0.54 points higher on our standardized scale of 1–10. Both lower and upper 95% CIs are above zero. The *I*^2^ value is 45%, indicating moderate heterogeneity [43]. As the paper by Maertens and colleagues [41] seemed to be an outlier, we explored what effect it would have on our results to exclude it from analysis. The standardized mean difference drops slightly to 0.42 (0.12 to 0.71, *P* = 0.005). However, *I*^2^ falls dramatically to 0% (Fig. 3b).

### Effect on operative time

Ten papers recorded operative times. Of these, two reported only median values and were excluded. One paper reported mean values, but failed to explicitly state if the accompanying values were ranges or 95% confidence intervals, and was thus excluded. One study presented only data analysis without reporting raw data or means; attempts were made to reconstruct these values without success. One study presented the total number of subjects enrolled, without mentioning the sizes of its control and intervention group. Attempts were made to calculate this given reported *P* values, but to no avail. Thus, a total of five studies had sufficient data quality to be included in our analysis of operative time differences.

**Fig. 4:**
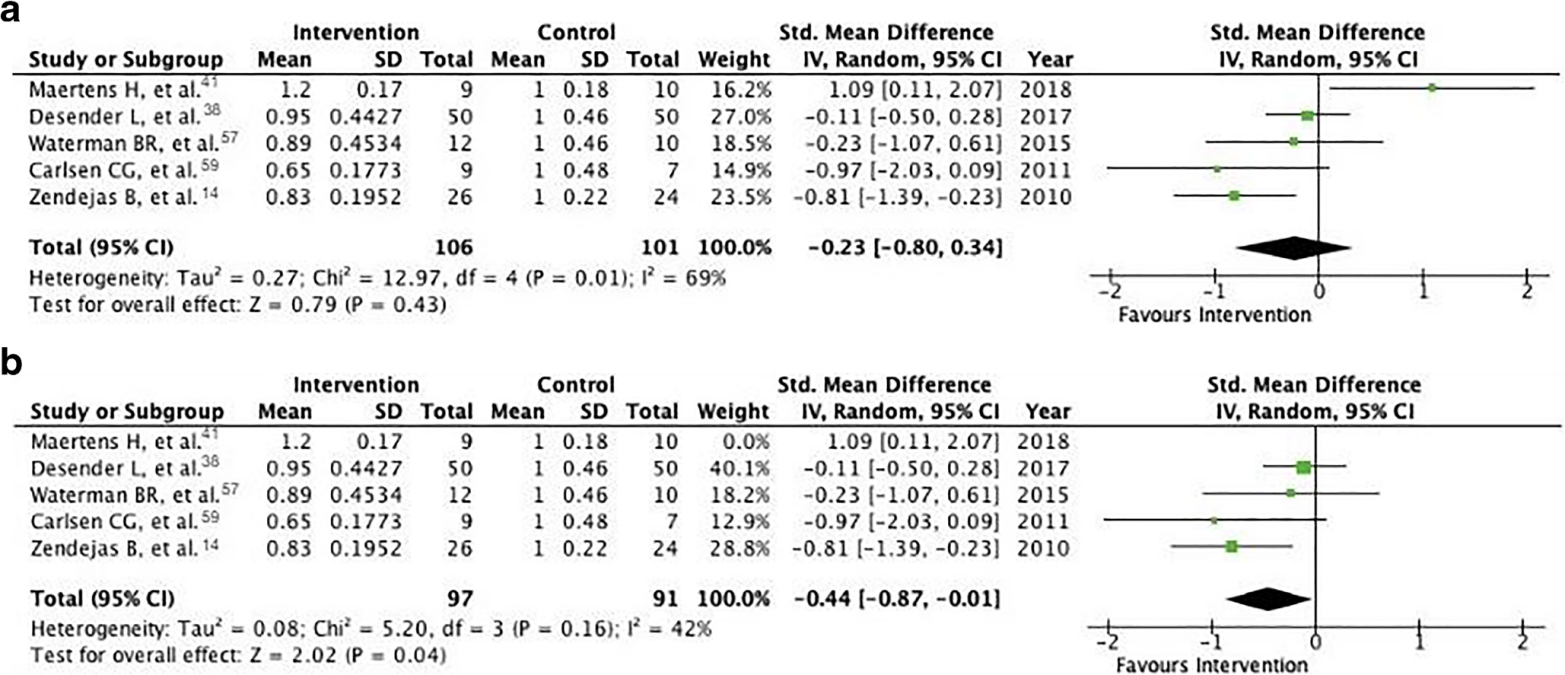
**a** Forest plot of the standardized mean difference of operative time between control and intervention groups, including tests for heterogeneity. **b** Forest plot of the standardized mean difference of operative time between control and intervention groups with outlier removed, including tests for heterogeneity

The standardized mean difference after simulator training was − 0.23 (− 0.80 to 0.34, *P* = 0.43) (Fig. 4a). Hence, the intervention group was on average 23% faster than the control group, but notably, the confidence interval is large and lies on both sides of 0. Heterogeneity is substantial [43], as indicated by an *I*^2^ value of 71%.

The study by Maertens and colleagues [41] was deemed to be an outlier (in the analysis of GRSs); thus, we again explored the effects of excluding it. Standardized mean difference becomes slightly larger with a value of − 0.44 (− 0.86 to − 0.01, *P* = 0.04). The confidence interval is slimmer and no longer intersects zero. Furthermore, heterogeneity is lower (*I*^2^ = 42%) (Fig. 4b).

## Discussion

Over the past few decades, there has been increasing interest in simulation technology in the field of surgery. There are multiple reasons for this, among them are technological advances that allow for increasing fidelity [53], residency work hour restrictions necessitating a shift of surgical education to outside ORs [9, 10, 12, 13, 54], and a changing medico-legal landscape concerning treatment standards [5]. However, the question is whether the enthusiasm in the field has translated to robust evidence regarding the benefits of simulation training.

In this review, using the PRISMA guidelines, we identified only 19 RCTs that investigated the impact of simulation training on the surgical treatment of patients. Study population size varied widely (range 3–73). Most of the procedures studied were endoscopic or laparoscopic in nature, and the enrolled subjects were predominantly residents (Table 1). Training primarily occurred on VR simulators, either for a fixed amount of time or until a predetermined level of proficiency was achieved (Table 2). However, only five papers established a performance baseline in the OR. Outcome measures in a clinical setting (not in a simulated environment) were diverse, but frequently, only a GRS of operative performance was used (Supplemental Digital Content File 4).

Study quality was mixed; only two trials appropriately randomized and blinded patients, and there were multiple instances of data collectors not being blinded (Table 3). Several papers had a high risk of selective reporting (Table 3). As such, our conclusions are tempered, but in line with previous systematic reviews and meta-analyses on the topic [55–57].

Thirteen of the studies found statistically significant effects of training on outcomes measured in the OR. Although these effects were deemed to be *potentially* clinically relevant, arguably, the most essential measure in surgery is patient outcome. Merely four of our included studies assessed the impact of simulation training on patient outcomes, and only one found a significant effect (Fig. 2).

With regard to meta-analyses, we synthesized the results of the overall effect of simulation training on operative performance. When standardized to a GRS scale from 1 to 10, participants who received simulation training scored an average of 0.42 points higher than their control group peers after intervention (*P* = 0.005) (Fig. 3b). Thus, there seems to be a positive effect of simulation training on performance as measured by a GRS, albeit a small one. Interestingly, chi^2^ and *I*^2^ tests of this analysis were low, indicating that this result was homogenous across included studies (Fig. 3b). Similarly, when operative times were standardized, simulation-trained participants were 44% faster (*P* = 0.04) (Fig. 4b), although heterogeneity of this analysis was moderate. This may in part be due to the inclusion of studies from different surgical fields; the difference in operative time of a novice compared with a master is unlikely to be the same across all types of procedures.

There are limitations to our study, prime among them being that few papers were included in qualitative analysis (*n* = 19), although we contend that this reflects the literature as a whole. As a result of diversity in recorded outcomes and stringent criteria for entry into data synthesis, our meta-analysis is based on an even smaller amount of studies (*n* = 6 for GRS and *n* = 4 for operative time). Furthermore, we included studies from various surgical fields, the results of which may not be comparable. Subgroup analyses were not conducted; we did not compare training for a given amount of time to training until reaching proficiency, or the effect of the training level of the study population on the effect of intervention, for example. Only one author was responsible for reviewing and excluding papers identified in our literature search, as well as for extracting data from included studies; this may have introduced bias to our findings.

Few systematic reviews have studied the impact of surgical simulation training on patient outcomes. Zendejas and colleagues found “small-moderate patient benefits,” although notably, their field of study was wider (all medical education) and patient outcomes were defined more broadly, including technical success of the procedure [56]. We chose not to include intraoperative errors and procedural success as patient outcomes, as they may be subject to a variety of interpretations (e.g., when is patient discomfort during a colonoscopy the result of poor scope movement and when is it an unavoidable result of strictures?) [58].

We found a positive effect of simulation training on achieved GRS score, as well as operative time; a finding that is echoed in the literature [55, 57, 59]. However, it must be noted that rating scales and operative time are surrogates of surgical proficiency and that technical surgical skills cannot be determined by one simple measure [57]. Scales such as the OSATS and OSA-LS have been thoroughly tested and show high inter-rater reliability [40], but are nevertheless based on subjective reflections made by observers [60]. Operative time may be a misleading metric; although expert surgeons perform procedures faster than residents [61], time at the cost of patient well-being is an unacceptable trade-off [62].

Whether or not the noted improvements are cost-effective requires further research. Aside from the initial expenses of necessary equipment, a host of other variables associated with implementation seem likely to be important. For example, can simulation training occur outside of work hours, or must it be added to existing schedules? Given the latter, do residents have spare time to accommodate this change or must it supplant other activities? Will simulation training come at the expense of time in the OR? Can training occur with an instructor? Crucially, the answers to these questions will not be identical across institutions, and as such, the efficacy of simulation training may vary.

To conclude, simulation training has a positive effect on OR performance and operative time, although there is little substantial evidence to date to support a direct beneficial effect on patient outcomes.

## Electronic supplementary material

Supplemental Digital Content File 1 . PDF. Study protocol. (PDF 29 kb)

Supplemental Digital Content File 2 . PDF. Screening. Results of the initial screening of 119 articles assessed for eligibility. (PDF 80 kb)

Supplemental Digital Content File 3 . PDF. CASP analysis. Detailed evaluation of 19 papers included in the study using the Critical Appraisal Skills Programme (CASP) - Randomized Controlled Trial (RCT) Checklist. (PDF 85 kb)

Supplemental Digital Content File 4 . PDF. Results. Detailed results of the 19 papers included in the study. (PDF 34 kb)

Supplemental Digital Content File 5 . PDF. Standardization of results. Detailed description of method to standardize outcome values across studies. (PDF 106 kb)
