# Supplementary material for: The impact of surgical simulation on patient outcomes: a systematic review and meta-analysis
Source: Neurosurg Rev. 2020 May 13;44(2):843–54. doi: 10.1007/s10143-020-01314-2 (PMC8035110; doi:10.1007/s10143-020-01314-2)
Supplement: Supplementary file 1 — . PDF. Study protocol. (PDF 29 kb) [file 10143_2020_1314_MOESM1_ESM.pdf]

Surgical Simulation Study Protocol

Evaluation of studies on simulation training will be performed according to the Preferred Reporting Items for Systematic Reviews and Meta-Analyses (PRISMA) statement, including the methods of publication search, eligibility, data collection, extraction, and analysis-, as well as preparation of the systematic review report.

A search of PubMed databases using the following criteria “surgery” [MeSH Terms] AND “simulation” [All Fields] AND “patient outcome” [All Fields] will be performed, including the following filters: English language, human species and randomized controlled trial article type. A PRISMA flow diagram will be created in order to visualize this process.

The papers identified from this search will be screened and the abstracts reviewed in order to determine relevance. Studies will be excluded if they do not involve surgery (e.g. vaginal child delivery), if the simulation described lacks involvement of manual skills (e.g. three-dimensional reconstruction of a cyst to plan surgery), if no patient treatments were conducted, or if all measurements were recorded in a simulated setting. The full text of the articles that remain will be reviewed, using the Critical Appraisal Skills Programme (CASP) - Randomized Controlled Trial (RCT) Checklist as a guide, with some additional items added by the authors.

Information sought in each paper will be: study population enrolled, study population in the final analysis, training level of study population, type of procedure studied, whether or not a OR baseline was established, use of intraoperative rating scale, recording of operative time, type of simulation used, time allotted to simulation training, type of training control group received, if the intervention group was significantly better than the control group after intervention, clinically relevant effects of training, if intervention group improved from its baseline following intervention and effect of intervention on patient outcomes.

Study quality will be assessed by evaluating the mode of randomization used, if the trial was stopped early, if patient assignment was truly randomized (e.g. assignment by random number generator or similar process), blinding of patients and data collectors, accounting of subjects at trial conclusion, evidence of selective reporting, and similarity of groups at the start of the trial.

We define patient outcomes as data or measurements of patients made after surgical intervention (in-hospital mortality, 30-day mortality, postoperative complications etc.). Operative time and subjective assessments of perioperative errors will not be considered patient outcomes.

Study data measured in a clinical setting (i.e. on real patients, not in a simulated environment) will be extracted by the first author and added to a data sheet. Meta analyses will be conducted on the effect of simulation training on performance as measured by a global rating scale (GRS) as well as operative time. To allow for the combination of data with respect GRS scores, an

arbitrary scale of 1-10 will used by authors; the GRS used by each study will then be mapped to this arbitrary scale and a standardized mean calculated. The following method will be used:

Standardizing Global Rating Scales

Using the following formulae:

Normal value = a + ((x-A)(b-a))/(B-A)

Where a = 1, b = 10, A = minimum value of trial rating scale, B = maximum value of trial rating scale, x = measured value of trial rating scale

Standard deviation, if 95% CI is given instead, calculated using:

Lower limit = mean - 1.96(SD/(n^0.5))
Upper limit = mean + 1.96(SD/(n^0.5))

Where n = number of participants in measured group

Rearranged

SD = ((n^0.5)(mean - lower limit))/1.96
SD = ((n^0.5)(upper limit - mean))/1.96

Then choosing whichever of the above gives the largest value of SD

Standard deviation normal = $[a + ((SD^2 - A)(b - a))(B - A)]$^0.5

To compare operative times, the mean result of the intervention group and that of the control group will be divided by the result of the control group. In this way, the control mean becomes 1 for all studies, and the intervention mean is expressed as a fraction of the control, with a value smaller or larger than 1, depending on whether the group was faster or slower. The following method will be used:

Expressing intervention procedure time as a fraction of the control intervention time

For all papers, control time, control time SD, intervention time and intervention time SD will be divided by the control time. Thus the intervention time is expressed as a fraction of the control time.

Standard deviation, if 95% CI is given instead, calculated using:

Lower limit = mean - 1.96(SD/(n^0.5))
Upper limit = mean + 1.96(SD/(n^0.5))

Where n = number of participants in measured group

Rearranged

SD = ((n^0.5)(mean - lower limit))/1.96
SD = ((n^0.5)(upper limit - mean))/1.96

Then choosing whichever of the above give the largest value of SD

If a study has more than two groups, the control group will be compared to the main intervention group. If groups were tested at multiple times, the results of the first test post-intervention will be used. In cases where a study used multiple rating scales, the most generic one will be chosen. If a paper reported 95% confidence intervals (CI), the standard deviation will be calculated. Papers that report results only as median values will be excluded from synthesis. In cases of incomplete data reporting, attempts will be made to calculate necessary values (e.g. from given P-values). If such attempts are unsuccessful, the study will be excluded from data synthesis. Lack of significant differences between intervention and control groups will not be a cause for exclusion from data synthesis. These actions will be observed in accordance with the Cochrane Collaboration's guidelines on conducting meta analyses outlined in Part 2, Chapter 9 of the Cochrane Handbook for Systematic Reviews of Interventions.

Data processing will be accomplished with the aid of Wolfram|Alpha. Meta-analyses will be performed using the Cochrane Collaboration's Review Manager software. Standardized means will be compared using an inverse-variance random-effects model. The effect size will be the standardized mean difference, Hedge's (adjusted) g. Heterogeneity will be assessed using the Chi^2 and I^2 tests.
