# Supplementary material for: The impact of surgical simulation on patient outcomes: a systematic review and meta-analysis
Source: Neurosurg Rev. 2020 May 13;44(2):843–54. doi: 10.1007/s10143-020-01314-2 (PMC8035110; doi:10.1007/s10143-020-01314-2)
Supplement: Supplementary file 3 — . PDF. CASP analysis. Detailed evaluation of 19 papers included in the study using the Critical Appraisal Skills Programme (CASP) - Randomized Controlled Trial (RCT) Checklist. (PDF 85 kb) [file 10143_2020_1314_MOESM3_ESM.pdf]

Supplemental Digital Content File 3. PDF. Detailed evaluation using the Critical Appraisal Skills Programme (CASP) - Randomized Controlled Trial (RCT) Checklist.

Paper Number	Final Paper Number	Authors	Notes	Cohort Size	Did the trial have a clearly focussed issue?	Was assignment of patients to treatments randomized?
4	1	Wooster M, et al.[27]	Carotid endovascular stenting, patients anatomy uploaded to simulator and practised on before surgery	15 (c=9, i=6)	Yes: does rehearsal using patient specific anatomy uploaded to a simulator improve procefural efficiency and outcomes?	Yes
8	2	Maertens H, et al.[41]	Endovascular interventions in lower extremity	32, 3 drouputs (c=10, i1=10, i2=9)	Yes: how does the PROSPECT training program compare to e-learning and traditional training with respect to acquisition of endovascular skills and the transferrability of these skills to real patient scenarios	Can't tell
11	3	Zevin B, et al.[56]	Bariatric surgery, practice and assessement on pigs, intervention group rated on live patient after trial	20 (c=10, i=10) additionally 9 chief residents	Yes: to develop and provide evidence of validity for a simulation-enhanced training curriculum for an advanced minimally invasive procedure	Can't tell
14	4	Desender L, et al.[29]	Aneurism repair, half of patients anatomy uploaded to simulator and practised on before actual operation	100 (c=50, i=50)	Yes: to evaluate the effect of patient specific rehearsal prior to endovascular aneurysm repair on patient safety and procedural efficiency	Yes
19	5	Nilsson C, et al.[17]	Simulation of endoscopic camera navigation. Simulation test and transfer test in OR, navigating camera in a cholecystectomy	36 (c=12, i= 11 (1 dropout), ii = 12)	Yes: how to train laparascopic camera navigation and the transfer of this training to the operating room	Can't tell
24	6	Waterman BR, et al.[57]	Diagnostic shoulder arthroscopy	22 (c=10, i=12)	Yes: does simulation training on diagnostic shoulder athroscopy improve task performance in the operating room	Can't tell
25	7	Shore EM, et al.[15]	Salpingectomy and intracorporeal knot tying, assessment in OR	27 (c=13 (2 excluded from primary outcome measure), i=14 (4 excluded from primary outcome measure))	Yes: to develop and validate a comprehensive ex-vivo training curriculum for gynecological laparoscopy	Can't tell
36	8	Patel NR, et al.[16]	Salpingectomy, porcine simulation model, human pre and post test	22 (c=11, i=11)	Yes: To evaluate the effectiveness of the porcine training model for OBGYN residents in laparoscopic salpingectomy	Can't tell
43	9	Dunn JC, et al.[58]	Arthroscopy og shoulder joint. In vivo test, then simulation, then another in vivo test. 1 year later new in vivo test to look at skill decay	17 (they legitimately dont report how many were in each group)	Yes: are gains made by residents after a simulation program retained after a period away from training?	Can't tell
46	10	Peltan ID, et al.[29]	Central venous catheter placement	73 (c=37, i=36), observed CVC placements=87 (c=38, i=49)	Yes: to evaluate the effects of a pragmatic CVC simulation program on procedural protocol adherence, technical skill and patient outcomes.	Can't tell
49	11	Grover SG, et al.[42]	Colonoscopy, simulation pre-test, training, post-test then in-vivo post-post-test	34, 1 droupout (c=17, i=16) control = self regulated learning, intervention = structured curriculum	Yes: to determine wether structured simulation based curriculum improves performance in colonoscopy and transfers to the clinical setting as compared to a self regulated curriculum.	Can't tell
53	12	Carlsen CG, et al.[59]	Inguinal hernia repair. Mannequin and pig simulation then in-vivo testing	18 (c=8, i=10), included in final analysis (c=7, i=9)	Yes: to test the effect of a module-based training model in Lichstenstein hernia repair rechnical skills	Can't tell
56	13	Koch AD, et al.[60]	Colonoscopy, both groups recieved VR training but different amounts	18 (i=8, ii=10) i=50 total, ii=100 total	Yes: to assess the clinical performance of novice endoscopists during colonoscopy after intensive and prolonged training on a VR endoscopy simulator	Can't tell
83	14	Zendejas B, et al.[14]	Laparascopic inguinal hernia repair	50 (c=24, i=26, crossover from control to intervention after TEP2=10)	Yes: to evaluate a mastery learning, simulation based curriculum for laparascopic, TEP inguinal hernia repair	Can't tell
85	15	Kessler DO, et al.[6]	Lumbar puncture on infants, self reported success after simulation training	56 (c=28, i=28) reported clinical LP (c=17, i=15) completed final OSCE assessment (c=25, i=26)	Yes: to demonstrate that deliberate practice simulation based training improves infant LP skills compared to a control group	Can't tell
91	16	Catalatayud D, et al.[30]	Warm-up in simulator or not before laparoscopic cholecystectomy	10 (8 actually analyzed in results)	Yes: to determine if a short VR warm-up curriculum improves laparoscopic performance in the OR	Can't tell
93	17	Haycock A, et al.[61]	Simulator training in colonoscopy	40 (c=20, i=20) included in final analysis (c=18, i=18)	Yes: the educational evaluation of the simulator as a tool for training novices in colonoscopy	Can't tell
101	18	Ahlberg G, et al.[2]	VR training in cholecystectomy	13 (c=6, i=7)	Yes: to assess the effect of proficiency-based VR training on the outcome of the first 10 entire cholecystectomies performed by novices	Can't tell
104	19	Cohen J, et al.[62]	Simulator training in colonoscopy	51, completed study 45 (c=23, i=22)	Yes: to define the benefit of training on the GI mentor on competency acquisition in colonoscopy	Can't tell

*See Data Sheet (Supplemental File 4) for treatment effect sizes and precision of estimated treatment effects
Columns F - P correspond to CASP Randomized Controlled Trial Checklist items 1 - 11

Were all of the patients who entered the trial properly accounted for at its conclusion?	Were patients/health workers/study personnel 'blind' to treatment?	Were the groups similar at the start of the trial?	Aside from experimental intervention, were the groups treated equally?	How large was the treatment effect?*	How precise was the estimate of the treatment effect?*
Can't tell	Authors claim observers were blinded, no mention of wether or not patients were blinded	Can't tell	Yes		
Yes	Non-blinded observer recorded operative metrics and consultant takeovers. Blinded consultant assessed GRS and examiner checklist after procedure. A blinded observer also assessed these post-hoc based on flouroscopy footage and hand movements.	Yes, with respect to sex, post-grad year and number of endovascular cases assissted	Yes, but no post testing of the control group was completed		
Can't tell	Porcine and OR performances assessed usings BOSATS scale by 1 trained and blinded rater. Non-technical skills assessed by NOTSS system by 2 trained raters, 1 blinded and 1 non blinded.	Yes, with respect to a host of variables, but intervention group had significantly fewer basic bariatric surgeries performed as the primary surgeon and bariatric rotations participated in	Yes, but the control group did perform the surgery being assessed on a human in the OR		
Yes	Can't tell if patients were blinded. Researchers assessing and enrolling patients, as well as outcome assessors were blinded.	Yes, inclusion criteria were age>18, non-rutputured aortic/iliac aneurism, suited to treatment with Gire Excluder AAA endoprosthesis/Endurant stent graft	Yes		
Can't tell	Can't tell	Yes with respect to age and experience with laparascopic training and surgery, but not sex	Yes		
Can't tell	Assessors were independent and blinded. Can't tell if patients were blind to treatment	Yes with respect to age, sex, post-grad year and arthroscopies performed pre and post intervention	Yes		
Can't tell	Assessors of operative performance were blinded. Assessment of pre--opertative non-technical skills was not blinded. Not clear if assessment on box trainer and simulator was blinded.	Yes with respect to a host of variables (surgical experience, VR experience, musical instrument experience etc.)	Yes		
Can't tell	Yes, single OSAT evaluater was blinded	Groups stratified by pre-intervention human salpingectomy OSAT score, post grad year was similar	Yes		
Can't tell	ASSET score assessed by 2 blinded evaluators. Can't tell if patients were blind to treatment	Yes with respect to sex, post-grad year and number of cases performed	Yes		
Yes	Supervisor (resident, fellow or attending) and the patients nurse rated the CVC placements and were blinded	Yes, with respect to age, sex, training track and degree	Yes		
Can't tell	JAG DOPS, Global Rating Form and Communication GRS were all blinded. Can't tell if patients were blind to treatment.	Yes, with respect to age, sex, training program and number of colonoscopies performed and assisted	Yes		
Can't tell	Assessors of surgical time and technical score were blinded	Yes, with respect to age, sex, time in surgical employement and prior number of performed hernia repairs	Yes, but control group was assed 2 times compared to the intervention group which was assessed 4 times		
Can't tell	Supervising surgeon was blinded to amount of VR simulated procedures the trainee had completed at the time of patient based assessment	Yes, all subjects were at the start of their training in gastroenterology with no previous endoscopic experience	Yes, but intervention group i completed 50 total VR colonoscopies and group ii completed 100 total VR colonoscopies		
Yes	Supervising staff surgeon was blind to the status of the resident surgeon, observer was not blinded (Staff GOALS score was used, both reviewed video recording to assess intraoperative complications). Retrospective assessment of patient medical records was done by a blinded member of staff.	Yes, baseline TEP repair was similar, groups were similar with respect to a host of other variables (post-grad year, sex, handedness, video game experience, TEP comfort + experience)	Yes, but the control group could cross over to the intervention protocol after TEP2, 10 participants elected to do this		
Can't tell	OSCE checklist assessors were not blinded, but 60% of the tests were assessed post hoc (video recording) by a blinded author.	Yes, with respect to sex, post-grad year and experience with LP (training, simulator experience, observations, LPs performed)	Yes		
Can't tell	OSATS score given by 2 blinded raters	Each surgeon served as their own control	Yes		
Can't tell	Expert assessors of patient colonoscopies were blinded	Yes, with respect to age, sex, educational direction, sigmoidoscopies and colonoscopies witnessed/assisted/performed	Yes		
Can't tell	Surgical supervisors were blinded. The 2 video assessors were blinded.	Yes, with respect to age, sex, visuospatial assessment, working memory assessment and laparascopic assisting experience	Yes		
Can't tell	Proctors who assessed fellows after the procedure were blinded	Yes, with respect to experience with gastroscopy and flexible sigmoidoscopies	Yes		

Can the results be applied to your context?	Were all clinically important outcomes measured?	Are the benefits worth the harms and costs?	What type of simulation was used?	How much time was allotted to simulation?
Yes	Measured outcomes: operative time, contrast volume, fluoroscopy time, time to carotid cannulation and carotid sheath duration	No significant effect demonstrated	ANGIO Mentor; Simbionix, Aiport City, Israel	As much as the primary operator wanted within 24 hours of the surgery
Yes	Measured outcomes: peri-operative complications, major + minor adverse events in hospital and 30 days after treatment, global rating scale, examiner checklist, supervisor takeovers, operative time, fluoroscopy time, DAP, number of angiograms and contrast volume	Cost effectiveness not assessed	ANGIO Mentor Express System; Simbionix, Cleveland, Ohio, USA	Time until proficiency, defined as passing an MCQ and passing technical proficiency on simulator 2 times at all 4 stages of the program. Mean time to completion was 8.44 months
Yes	Measured outcome: BOSATS score	No significant effect demonstrated	Laparascopic box trainer using cadaveric porcine small bowel	Time until proficiency, defined as achieving a BOSATS score of 80% or more
Yes	Measured outcomes: peri-operative errors, technical + clinical success rates, in-hospital + 30 day mortality, global rating scale score, procedure specific rating scale score, surgical + nursing team OTAS score, operative time, flouroscopy time, contrast volume, number of angiograms until deployment of main body, number of angiograms until deployment of all stent grafts, total number of angiograms, radiation dose (DAP)	Cost effectiveness not assessed	ANGIO Mentor Express Dual Access Simulation System; Simbionix, Cleveland, Ohio, USA	Primary operator, assisstant and scrub nurse performed pre-operative rehearsal 1 time within 24 hours of the actual surgery
Yes	Measured outcome: OSA-CNS score	No significant effect demonstrated	LapSim; Surgical Science, Gothenburg, Sweden	2 hours
Yes	Measured outcomes: ASSET score, Safety score, Anatomic checklist and operative time	Cost effectiveness not assessed	Arthro VR shoulder simulator, Simbionix, Cleveland, Ohio, USA	1 hour
Yes	Measured outcomes: OSA-LS score, residents who completed intracorporeal knots, knot tying global rating scale score and time required to complete knot	Cost effectiveness not assessed	LapSim; Surgical Science, Gothenburg, Sweden. Box trainers for peg transfer, pattern cutting, pretied knot, ovarian cystectomy, Roeder knots and intracorporeal knot tying (which served as the post test). SimMan; Laerdal Medical, Stavanger, Norway (single simulation of CO embolus during laparoscopy to train non-techinical skills in the intervention group)	Seven 2 hour sessions on box trainer and VR simulator. Access to these was not restricted to either group outside of the mandatory sessions. 1 session for intervention group on non-technical skills 10-15min.
Yes	Measured outcome: OSAT score	Cost effectiveness not assessed	Porcine cadavers	1 hour to familiarize, 0.5 hours after to do 1 surgery
Yes	Measured outcomes: ASSET score, ASSET safety score, operative time and anatomical checklist score	No significant effect demonstrated	Arthro VR shoulder simulator, Simbionix, Cleveland, Ohio, USA	1 hour
Yes	Measured outcomes: complications (arterial puncture, hematoma, catheter malposition, catheter-associated infection, pneuthorax and death), first-pass attempt success, overall cannulation success, mean needle passes required, global assessment score and average procedural protocol adherance	Cost effectiveness not assessed	Venous acces simulator, Blue Phantom, Redmond, Washington, USA	1-2 hours of individual training, then training as needed to pass independent CVC placement
Yes	Measured outcome: JAG DOPS score	Cost effectiveness not assessed	EndoVR, CAE Healthcare Canada, Quebec, Montreal, Canada	8 hours of practice on simulators (control did not get instruction or feedback from professional)
Yes	Measured outcomes: global rating scale score and duration of procedure	Cost effectiveness not assessed	"plastic phantom mimicking the human male groin" and surgery of anesthetized pig with congenital hernia	1 day skills lab course with pracite on plastic phantom and anesthetized pig
Yes	Measured outcomes: depth of insertion and number of cecal intubations achieved	Cost effectiveness not assessed	GI Mentor II, Simbionix, Cleveland, Ohio, USA	Amount of completed VR cases, group i was tested twice in patient after 10, 30 and 50 VR cases completed, group ii was tested twice in patients after 20, 60 and 100 VR cases completed.
Yes	Measured outcomes: intraoperative complications (vessel + bladder injury, peritoneal tear, conversion of surgical approach (open or trans-abdominal)), post-op complications (hematoma, seroma, skin infection and urinary retention), post-post-op complications (overnight stay, recurrence of hernia and groin pain 3-months post repair), operative time, adjusted operative time, proportion of procedure completed by resident and GOALS score	Cost effectiveness not assessed	Guildford MATTU TEP hernia task trainer; Limbs and Things, Bristol, UK	Simulation practice until mastery was achieved, defined as successful repair of both hernias (bilateral) in less than 2 minutes on 2 consecutive attempts.
Yes	Measured outcomes: success rate, rate of traumatic intervention and number of attempts	Cost effectiveness not assessed	Baby Stap neonatal task trainer; Laerdal, Wappinger Falls, New York, USA)	Simulation practice until mastery was achieved, defined as demonstrating all steps (15 point checklist) flawlessly and independently from start to finish on the simulator.
Yes	Measured outcome: OSATS score	Cost effectiveness not assessed	LapSim; Surgical Science, Gothenburg, Sweden	3 laparascopic tasks (7 available at 3 different difficulty levels), lasting approx. 15min
Yes	Measured outcomes: JAG DOPS score, Global Score, completion rate, maximum tip position reached, operative time and straight insertion depth	No significant effect demonstrated	Endo TS-1; Olympus Keymed, Southerland, UK	Intervention group recieved 16 hours of practice on simulator. Control group recieved 16 hours of practice on patients (4 half day sessions, required to perform a minimum of 8 procedures per session)
Yes	Measured outcomes: operative time, total errors, erros in exposure + clipping and tissue division + dissection (parameters for each such as lack of progress, structure injury, attending takeover etc.)	Cost effectiveness not assessed	LapSim; Surgical Science, Gothenburg, Sweden	Intervention group recieved simulator training until they achieved proficiency, defined as achieving the median score of 5 expert laparoscopists across 6 tasks twice
Yes	Measured outcomes: subjective patient discomfort, subjective competence, objective competence and number of cases needed to reach 95% subjective and objective competence	Cost effectiveness not assessed	GI Mentor, Simbionix, Cleveland, Ohio, USA	Intervention group practiced on the VR simulator for 10 hours prior to performing live colonoscopies

Control group training	Which patient outcomes were recorded?	Further notes
None	None	15 patients over 3 years, 2 hospitals. Not really any patient outcomes other that duration of procedure and contrast volume used. Unclear how similar the groups were. Unclear who was assessing the surgeries. Complete and utter lack of statistical power.
Continued conventional training	Recorded patient outcomes: peri-operative complications, major + minor adverse events in hospital and 30 days after treatment	2 live surgeries after intervention. Statistical improvement in GRS, Examiner Checklist and nr of takeovers. No differences aside from these. Authors argue consultants taking over may have prevented differences in patient outcomes. Unclear if groups were balanced.
Continued conventional training	None	Intervention with training on box trainer with porcine cadaver lead to better psychomotor performance on anesthetized porcine model than peers who recieved standard training. Psychomotor performance by the tested 3. year residents was equivalent to that of Chief Residents (8/10 in intervention group allowed, only part of surgery, only 1 surgery). No patient outcomes recorded.
Rehearsal after procedure	Recorded patient outcomes: peri-operative errors, technical + clinical success rates, in-hospital + 30 day mortality	Rehearsing led to changes in the pre-op plan (88% changed, 92% implemented) and led to fewer perioperative mistakes. However there was no statistically significant improvement to technical skills (already proficient teams) or patient mortality (equally low for both groups)
None	None	Control, practice on camera navigation and practice on actual procedure. Camera navigation performed better on simulation test (different to the simulation training). No statistical differences between the groups upon transfer to the OR.
Continued conventional training	None	1 hour of simulator practice lead to better time, but otherwise no statistically significant improvements compared to regular training. Patients were not randomized. No patient outcomes recorded.
Continued conventional training	None	Seven 2 hour practice sessions on box trainer and VR simulator. Technical performance assessed with OSA-LS was better in intervention group. Intracorporeal knot tying was not statistically different. No patient outcomes measured.
Continued conventional training	None	Improvement to 2/9 items on OSAT and overall OSAT after 1.5 hours practice on porcine cadavers. Overall improvement from 26.7 to 29.9 (3 points), which the authors note is little. Both groups performed poorer than would be expected (c=26.2 and i=29.9 out of 45 possible points)
Continued conventional training	None	Initial testing on patient, then 1 hour of sim practice for intervention group, then re-testing on patient, then re-testing on patients after 1 year. Intervention group had statistical improvements in ASSET score and time to completion from test 1 to 2. No other significant differences. Time incrased between test 2 and 3 by 18% (mean), so improvements were not retained after 1 year.
Continued conventional training	Recorded patient outcomes: arterial puncture, hematoma, catheter malposition, catheter-associated infection, pneuthorax and death	Simulation training untill achieved mastery. Intervention group had significantly better adherence to procedural protocol. All other measured variables were similar between the groups. Problem with the study is few observed CVC placements (87) and the hospital already having an excellent track record. Observer rated placement that they themselves supervised. Supervisor would step in if necessary (was noted in GRS)
Simulation training without feeback	None	8 hours of practice on sim, sim test before intervention, right after then 4-6 weeks after. Clinical colonoscopy 4-6 weeks after intervention. Intervention group was statistically better than control group with respect to JAG DOPS score at clinical colonoscopies. No patient outcomes measured. Highlights difference between running sim practice alone and getting feedback from expert.
Continued conventional training	None	Intervention group recieved 1 day of skills training on plastic phantom and anesthetized pig. They then completed 20 hernia repairs over the course of the 4-6 days. The control group had standard training, doing hernia repairs whenever the department deemed it to be apppropriate. Both groups were compared at the end of the year (the first year of surgical specialty training). Intervention led to statistically better technical score and time to completion, these improvements were retained (but not improved on) at the end of the year. intervention group had significantly better technical scores at the end of the year compared to control group, but not time to completion (better, but p=0.059). No clinically important outcomes measured.
Number of procedures**	None	Both groups recieved sim traning, group i for a total of 50 VR cases and group ii for a total of 100 VR cases. Improvement on sim plateued after 60 VR cases, improvement in patients plateued after 50 VR cases. Plateu = no further statistically significant improvements. Highlights diminishing returns of VR sim practice.
Continued conventional training	Recorded patient outcomes: intraoperative complications (vessel + bladder injury, peritoneal tear, conversion of surgical approach (open or trans-abdominal)), post-op complications (hematoma, seroma, skin infection and urinary retention), post-post-op complications (overnight stay, recurrence of hernia and groin pain 3-months post repair)	Intervention group trained on simulator until they achieved mastery. At TEP2 the intervention group was faster, completed more of the procedure themselves and had lower rates of complications. At TEP3 the residents who crossed over from control to intervention performed faster than their control counterparts. At TEP3/4/5 GOALS scores were not significantly different between the groups. Complications were similar between crossover and control groups at TEP3. All TEPs after intervention combined, and excluding the crossover group, the intervention group were statistically better in all measured outcomes (aside from overnight stays). Seems like a slam dunk. Why is crossover and control similar at TEP3, does that not mean intervention and control are similar at TEP3? Difference in total measures is significant but groups compared at TEP3/4/5 isnt?
Continued conventional training	None	Intervention group trained on simulator until mastery. Thereafter self reported success rate of first infant LP they perfomed. Intervenetion group was significantly more successful than the control group (95% vs 47%, P=0.005) and had fewer traumatic procedures (but not significantly fewer). Self reported success, very small sample size, at 6 months 9 of the 20 who said they had not perfomed an LP admitted that they had.
Surgeons served as there own controls	None	8 surgeons completed 2 surgeries each, 1 with warm up before and 1 without. Surgeries with warm-up beforehand recieved significantly better OSATS scores. No other variables measured. Small sample size.
16 hours of practice on patients	None	Intervention group recieved 16 hours of sim practice, control group recieved 16 hours of practice on patients. At post intervention assessment the intervention group outperformed control on the simultor. There were no statistically significant differences between groups upon patient based assessment. Interesting because it compares patient and sim practice directly.
Continued conventional training	None	7 trainees in the intervention group, performed better in their first 10 cholecystectomies (assessed at 1+5+10). They were 58% faster (barely not significant) and the control group made 3x more mistakes. Interesting to note that neither one of the groups improved from surgery 1 to 10. There was substabtially more variability in the control group (8x more variability in total errors in control group compared to intervention)
Continued conventional training	None	23 subjects performed 10 hours of practice on a VR simulator. Unclear how similar the groups were at the start. Subjective discomfort was similar between the groups. Objective competence was significantly better in the intervention group, but this effect tapered off and was no longer significant after 80 procedures. Subjective competence was significantly better in the intervention group until 40 performed procedures. Both groups improved over time, more at the start than at the end. Both groups needed the same amount of median cases to reach competency. Highlights that not all residents become competent at the same rate. The intervention group was “ahead on the learning curve” compared to control at several points in time.
