# Supplementary material for: The impact of surgical simulation on patient outcomes: a systematic review and meta-analysis
Source: Neurosurg Rev. 2020 May 13;44(2):843–54. doi: 10.1007/s10143-020-01314-2 (PMC8035110; doi:10.1007/s10143-020-01314-2)
Supplement: Supplementary file 4 — . PDF. Results. Detailed results of the 19 papers included in the study. (PDF 34 kb) [file 10143_2020_1314_MOESM4_ESM.pdf]

Supplemental Digital Content #4. PDF. Detailed results of studies included.

Authors	Measured Outcome	Control group baseline	Training group baseline	Third group baseline	Control group after intervention	Training group after intervention	Third group after intervention	Control group after intervention and delay	Training group after intervention and delay
Wooster M, et al.[27]*	Contrast volume (mL)				Mean 76.9; Range 38.0-120.0	Mean 59.2; Range 29.0-108.0			
	Fluoroscopy time (min)				Mean 19.4; Range 9.8-38.0	Mean 11.4; Range 5.4-19.8			
	Operative time (min)				Mean 42.5; Range 30.9-69.0	Mean 31.9; Range 14.2-54.0			
	Time to carotid cannulation (min)				Mean 23.3; Range 13.4-30.5	Mean 17.0; Range 7.2-30.1			
	Carotid sheath duration (min)				Mean 19.2; Range 11.8-26.5	Mean 14.9; Range 6.1-23.9			
Maertens H, et al.[41]	Global rating scale (0-48)				Mean 23.1; SD 2.2	Mean 39.4; SD 2.1	Mean 28.4; SD 2.2		
	Examiner checklist (0-72)				Mean 38.7; SD 3.4	Mean 63.5; SD 3.2	Mean 53.6; SD 3.3		
	Supervisor take-overs				Mean 4.2; SD 0.5	Mean 0.3; SD 0.5	Mean 3.4; SD 0.5		
	Operative time (min)				Mean 42.6; SD 7.5	Mean 51.3; SD 7.1	Mean 53.2; SD 7.5		
	Fluoroscopy time (min)				Mean 11.4; SD 1.5	Mean 12.0; SD 1.4	Mean 14.4; SD 1.5		
	DAP				Mean 61.8; SD 0.4	Mean 44.1; SD 3.8	Mean 61.3; SD 0.0		
	Number of angiograms				Mean 10.9; SD 3.5	Mean 9.0; SD 3.3	Mean 15.0; 3.4		
	Contrast volume (mL)				Mean 71.4; SD 9.7	Mean 65.1; SD 9.2	Mean 78.8; 9.6		
	Peri-operative complications (0-52)				2/52 (3.5%)	2/52 (3.5%)	4/52 (6.7%)		
	In-hospital minor adverse effects (0-58)				0/58 (0.0%)	2/52 (3.5%)	1/58 (1.7%)		
	In-hospital major adverse effects (0-58)				1/58 (1.7%)	1/58 (1.7%)	1/58 (1.7%)		
	30-day minor adverse effects (0-58)				3/58 (3.5%)	0/58 (0.0%)	1/58 (1.7%)		
	30-day major adverse effects (0-58)				1/58 (1.7%)	0/58 (0.0%)	0/58 (0.0%)		
Zevin B, et al.[56]	BOSATS score					Median 63; IQR 61-68			
Desender L, et al.[29]	Global rating scale (7-35)				Median 28; IQR 26-31	Median 29; IQR 26-31			
	Procedure specific rating scale (7-35)				Median 26; IQR 23-29	Median 27; IQR 24-30			
	Observational teamwork assessment for surgery tool, surgical team (0-30)				Median 17; IQR 12-22	Median 19; IQR 16-24			
	Observational teamwork assessment for surgery tool, nursing team (0-30)				Median 18; IQR 15-22	Median 18; IQR 14-22			
	Operative time (min)				Mean 54.6; 95% CI 48.4-61.6	Mean 52.1; 95% CI 46.2-58.8			
	Fluoroscopy time (sec)				Mean 864; 95% CI 720-1037	Mean 916; 95% CI 763-1099			
	Contrast volume (mL)				Mean 93; 95% CI 84-104	Mean 81; 95% CI 73-91			
	Number of angiograms until deployment of main body				Mean 2.8; 95% CI 2.5-3.2	Mean 2.2; 95% CI 1.9-2.4			
	Number of angiograms until deployment of all stent grafts				Mean 5.4; 95% CI 4.8-6.0	Mean 4.3; 95% CI 3.8-4.8			
	Total number of angiograms				Mean 7.5; 95% CI 6.7-8.2	Mean 6.5; 95% CI 5.9-7.2			
	Radiation dose, DAP (mGycm^2)				Mean 112.9; 95% CI 86.5-147.4)	Mean 104.0; 95% CI 79.7-135.7			
	Minor errors, complete procedure				Mean 4.2; 95% CI 3.7-4.9	Mean 3.1; 95% CI 2.7-3.7			
	Minor errors, edovascular part				Mean 3.8; 95% CI 3.3-4.4	Mean 3.0; 95% CI 2.6-3.5			
	Major errors, complete procedure				Mean 0.3; 95% CI 0.2-0.6	Mean 0.1; 95% CI 0.0-0.2			
	Major errors, endovascular part				Mean 0.2; 95% CI 0.1-0.4	Mean 0.0; 95% CI 0.0-0.2			
	Total errors, endovascular part				Mean 4.0; 95% CI 3.5-4.6	Mean 3.1; 95% CI 2.6-3.6			
	Total errors, non-endovascular part				Mean 0.5; 95% CI 0.4-0.8	Mean 0.2; 95% CI 0.1-0.3			
	Errors without delay				Mean 1.2; 95% CI 0.9-1.6	Mean 0.8; 95% CI 0.6-1.1			
	Errors causing delay				Mean 3.4; 95% CI 2.9-3.9	Mean 2.5; 95% CI 2.1-2.9			
	Technical succes, primary				39/50 (78%)	41/50 (82%)			
	Technical succes, assisted primary				43/50 (86%)	47/50 (94%)			
	Technical succes, secondary				46/50 (92%)	47/50 (94%)			
	Clinical success, primary				49/50 (98%)	45/50 (90%)			
	Clinical success, assisted primary				49/50 (98%)	47/50 (94%)			
	Clinical success, secondary				49/50 (98%)	48/50 (96%)			
	In-hospital mortality (0-50)				1/50 (2%)	0/50 (0%)			
	30-day mortality (0-50)				1/50 (2%)	2/50 (4%)			
Nilsson C, et al.[17]	OSA-CNS (5-25)				Mean 14.3; 95% CI 11.9-16.6	Mean 14.0; 95% CI 11.9-16.1	Mean 12.3; 95% CI 10.7-14.0		
Waterman BR, et al.[57]	ASSET (8-38)	Mean 19.1; SD 1.5	Mean 19.3; SD 1.5		Mean 21.3; SD 1.5	Mean 22.5; SD 1.5			
	ASSET safety (1-5)	Mean 3.0; SD 0.2	Mean 2.9; SD 0.2		Mean 3.0; SD 0.2	Mean 3.3; SD 0.2			
	Anatomic checklist (0-14)	Mean 11.0; SD 1.7	Mean 10.7; SD 3.7		Mean 11.4; SD 2.3	Mean 11.2; SD 3.2			
	Operative time (sec)	Mean 297.3; SD 153.6	Mean 294.7; SD 127.8		Mean 232.0; SD 106.0	Mean 205.9; SD 105.2			
Shore EM, et al.[15]	OSA-LS (10-50)				Median 30.0; IQR 27-35	Median 34.0; IQR 32.3-29.3			
	Completed intracorporeal knot				5/11 (45.5%)	8/10 (80%)			
	Knot tying global rating scale (5-20)				Median 12.0	Median 12.5			
	Time required to complete knot (sec)				Median 450.0	Median 427.5			
Patel NR, et al.[16]	OSATS (9-45)	Mean 26.6; SD 10.8	Mean 26.7; SD 10.6		Mean 26.2; SD 10.1	Mean 29.9; SD 9.8			
Dunn JC, et al.[58]	ASSET (8-38)	Mean 19.1; SD 6.6	Mean 19.3; SD 5.7		Mean 21.2; SD 6.6	Mean 22.5; SD 7.2		Mean 21.2; SD 1.1	Mean 21.5; SD 2.1
	ASSET safety (1-5)	Mean 2.9; SD 0.9	Mean 2.9; SD 0.8		Mean 3.0; SD 0.9	Mean 3.3; SD 1.1		Mean 2.8; SD 0.7	Mean 3.0; SD 0.0
	Anatomic checklist (0-14)	Mean 11.0; SD 1.7	Mean 10.7; SD 3.4		Mean 11.4; SD 1.0	Mean 11.2; SD 3.2		Mean 12.7; SD 1.0	Mean 11.7; SD 1.3
	Operative time (min)	Mean 5.0; SD 2.6	Mean 4.9; SD 2.1		Mean 3.9; SD 1.8	Mean 3.4; SD 1.8		Mean 3.6; SD 1.2	Mean 4.0; SD 1.5
Peltan ID, et al.[29]	Cannulation success on first attempt				23/38 (60.5%)	29/49 (59.2%)			
	Overall cannulation success				34/38 (89.5%)	45/49 (91.8%)			
	Needle passes required				Mean 1.64; SD 1.11	Mean 1.57; SD 1.02			
	Global assessment score (1-5)				Mean 2.9; SD 1.1	Mean 3.1; SD 1.1			
	Arterial puncture				5,4%	4,1%			
	Hematoma				5,3%	10,2%			
	Catheter malposition				6,1%	11,4%			
	Cather associated infection				3,2%	2,6%			
	Pneumothorax				0,0%	0,0%			
	Death				0,0%	0,0%			
	Average procedural protocol adherence				Mean 93%; 95% CI 90%-97%	Mean 82%; 95% CI 74%-91%			
Grover SG, et al.[42]**	JAG DOPS				NA	NA			
Carlsen CG, et al.[59]	Global rating scale (8-40)	Mean 23.4; 95% CI 19.4-27.3	Mean 22.5; 95% CI 20.6-24.3		Mean 21.7; 95% CI 17.3-26.1	Mean 26.9; 95% CI 23.4-30.4			
	Operative time (min)	Mean 52.3; 95% CI 39.3-65.3	Mean 53.3; 95% CI 45.7-60.9		Mean 61.3; 95% CI 39.3-83.3	Mean 40.1; 95% CI 33.0-47.2			
Koch AD, et al.[60]	Insertion depth (cm)	Mean 29.4; 95% CI 22.6-36.2	Mean 36.5; 95% CI 30.3-42.7		Mean 38.6; 95% CI 31.4-46.1	Mean 60.5; 95% CI 51.8-69.1		Mean 58.5; 95% CI 50.9-66.1	Mean 63.7; 95% CI 55.1-72.2
	Number of cecal intubations	0	0		1	1		1	3
Zendejas B, et al.[14]	Operative time, raw (min)	Mean 40.5; SD 8.4	Mean 41.5; SD 7.0		Mean 37.4; SD 8.3	Mean 30.9; SD 7.3		Mean 35.7; SD 7.6	Mean 29.6; SD 6.7
	Operative time, adjusted for participation (min)	Mean 53.5; SD 14.6	Mean 54.5; SD 11.8		Mean 47.5; SD 13.9	Mean 34.4; SD 8.4		Mean 44.2; SD 12.9	Mean 32.4; SD 7.7
	Resident participation	Mean 68%; SD 18%	Mean 69%; SD 16%		Mean 73.7%; SD 16.4%	Mean 88.4%; SD 9.4%		Mean 77.1%; SD 16.2%	Mean 90.8%; SD 8.3%
	GOALS (6-30)	Mean 17.4; SD 4.0	Mean 17.5; SD 3.8		Mean 18.3; SD 3.8	Mean 21.9; SD 2.7		Mean 18.7; SD 3.8	Mean 22.3; SD 3.0
	Peritoneal tear	4/36 (11.1%)	4/40 (10%)		9/37 (24.3%)	1/38 (2.6%)		13/58 (22.4%)	4/72 (5.6%)
	Epigastric vessel injury	1/36 (2.78%)	0/40 (0%)		4/37 (10.8%)	1/38 (2.6%)		4/58 (6.9%)	1/72 (1.4%)
	Bladder injury	NA	NA		1/37 (2.7%)	0/38 (0.0%)		1/58 (1.7%)	0/72 (0%)
	Conversion TAPP	NA	NA		1/37 (2.7%)	0/38 (0.0%)		1/58 (1.7%)	0/72 (0%)
	Urinary retention	7/36 (19.4%)	3/40 (7.5%)		9/37 (24.3%)	0/38 (0.0%)		12/58 (20.7%)	3/72 (4.2%)
	Seroma	NA	NA		0/37 (0.0%)	0/38 (0.0%)		1/58 (1.7%)	0/72 (0%)
	Hematoma	2/36 (5.56%)	0/40 (0%)		2/37 (5.4%)	1/38 (2.6%)		0/58 (0%)	1/72 (1.4%)
	Superficial skin infection	0/36 (0%)	1/40 (2.5%)		2/37 (5.4%)	0/38 (0.0%)		2/58 (3.4%)	0/72 (0%)
	Overnight stay	6/36 (16.7%)	3/40 (7.5%)		9/37 (24.3%)	0/38 (0.0%)		12/58 (20.7%)	5/72 (6.9%)
	Recurrence of hernia	NA	NA		0/37 (0.0%)	1/38 (2.6%)		NA	NA
	Groin pain 3 months post-repair	NA	NA		1/37 (2.7%)	1/38 (2.6%)		NA	NA
Kessler DO, et al.[6]	Obtained CSF				7/15 (47%)	16/17 (94%)			
	CSF < 1000 RBCs				4/15 (27%)	12/17 (71%)			
	Number of attempts				Median 1; IQR 0.75	Median 1; IQR 1			
Catalatayud D, et al.[30]	OSATS (5-35)				Median 19.3; Range 15.0-31.5	Median 28.5; Range 18.5-32.0			
Haycock A, et al.[61]	Completion of case				4/54 (7%)	6/54 (11%)			
	Maximum tip position: sigmoid				28/54 (52%)	29/54 (54%)			
	Maximum tip position: descending				12/54 (22%)	8/54 (15%)			
	Maximum tip position: transverse				8/54 (15%)	11/54 (20%)			
	Maximum tip position: ascending				2/54 (4%)	0/54 (0%)			
	Maximum tip position: coecum				4/54 (7%)	6/54 (11%)			
	Operative time (min)				Median 20; IQR 20-20	Median 20; IQR 19-20			
	Straight insertion depth (cm)				Mean 52; SD 21	Mean 48; SD 23			
	JAG DOPS (8-36)				Median 18; IQR 14-21	Median 16; IQR 14-22			
	Global Rating Scale (7-35)				Median 17; IQR 14-19	Median 16; IQR 14-19			
Ahlberg G, et al.[2]	Total errors				Mean 86.2; 95% CI 58.2-114.1	Mean 28.4; 95% CI 23.5-33.3			
	Exposure errors				Mean 53.4; 95% CI 16.7-90.1	Mean 15.0; 95% CI 11.2-18.8			
	Clipping and tissue division errors				Mean 7.1; 95% CI 4.0-10.3	Mean 1.9; 95% CI 0.9-2.9			
	Dissection errors				Mean 29.5; 95% CI 14.0-45.0	Mean 11.5; 95% CI 8.8-14.1			
	Operative time (min)				NA	NA			
Cohen J, et al.[62]	Number of cases needed to reach 95% objective competence				Median 8; 95% CI 8-9	Median 8; 95% CI 8-9			
	Number of cases needed to reach 95% subjective competence				Median 7; 95% CI 6-8	Median 7; 95% CI 6-8			
	Objective competence at session 1				Mean 40.9	Mean 50.4			
	Objective competence at session 2				Mean 52.0	Mean 64.5			
	Objective competence at session 3				Mean 62.0	Mean 74.0			
	Objective competence at session 4				Mean 64.4	Mean 76.7			
	Objective competence at session 5				Mean 70.2	Mean 76.8			
	Objective competence at session 6				Mean 77.6	Mean 77.8			
	Objective competence at session 7				Mean 80.5	Mean 80.8			
	Objective competence at session 8				Mean 83.7	Mean 89.5			
	Objective competence at session 9				Mean 85.2	Mean 87.8			
	Objective competence at session 10				Mean 90.9	Mean 92.7			
	Subjective competence at session (1-5) 1				Mean 36.6	Mean 47.6			
	Subjective competence at session (1-5) 2				Mean 57.4	Mean 68.6			
	Subjective competence at session (1-5) 3				Mean 68.4	Mean 76.3			
	Subjective competence at session (1-5) 4				Mean 75.4	Mean 78.0			
	Subjective competence at session (1-5) 5				Mean 79.4	Mean 81.3			
	Subjective competence at session (1-5) 6				Mean 82.3	Mean 82.0			
	Subjective competence at session (1-5) 7				Mean 84.1	Mean 86.1			
	Subjective competence at session (1-5) 8				Mean 86.4	Mean 88.8			
	Subjective competence at session (1-5) 9				Mean 86.8	Mean 88.9			
	Subjective competence at session (1-5) 10				Mean 90.5	Mean 90.8			
	Patient discomfort at session (5-1) 1				Mean 31.4	Mean 25.7			
	Patient discomfort at session (5-1) 2				Mean 19.1	Mean 23.2			
	Patient discomfort at session (5-1) 3				Mean 19.5	Mean 16.7			
	Patient discomfort at session (5-1) 4				Mean 18.2	Mean 16.0			
	Patient discomfort at session (5-1) 5				Mean 16.5	Mean 16.7			
	Patient discomfort at session (5-1) 6				Mean 13.9	Mean 13.4			
	Patient discomfort at session (5-1) 7				Mean 11.3	Mean 11.9			
	Patient discomfort at session (5-1) 8				Mean 10.4	Mean 10.5			
	Patient discomfort at session (5-1) 9				Mean 11.8	Mean 10.7			
	Patient discomfort at session (5-1) 10				Mean 9.2	Mean 8.9			

*Paper authors do not make clear if values presented were mean (IQR), mean (95% CI) or mean (range). We assumed for the completeness of the table that values were mean (range)

**JAG DOPS raw data or mean/median/mode values not provided, only figures and analysis of differences between the groups
