# Supplementary material for: The impact of surgical simulation on patient outcomes: a systematic review and meta-analysis
Source: Neurosurg Rev. 2020 May 13;44(2):843–54. doi: 10.1007/s10143-020-01314-2 (PMC8035110; doi:10.1007/s10143-020-01314-2)
Supplement: Supplementary file 5 — . PDF. Standardization of results. Detailed description of method to standardize outcome values across studies. (PDF 106 kb) [file 10143_2020_1314_MOESM5_ESM.pdf]

Data Processing

Standardizing Global Rating Scales

Using the following formulae:

Normal value = a + ((x-A)(b-a))/(B-A)

Where a = 1, b = 10, A = minimum value of trial rating scale, B = maximum value of trial rating scale, x = measured value of trial rating scale

Standard deviation, if 95% CI is given instead, calculated using:

Lower limit = mean - 1.96(SD/(n^0.5))
Upper limit = mean + 1.96(SD/(n^0.5))

Where n = number of participants in measured group

Rearranged

SD = ((n^0.5)(mean - lower limit))/1.96
SD = ((n^0.5)(upper limit - mean))/1.96

Then choosing whichever of the above give the largest value of SD

Standard deviation normal = $\left[a \ + \ ((SD\hat{}2 - A)(b-a))(B-A)\right]$ ^0.5

Maertens H, et al.[41]:

Scale is 0-48, A = 0 B = 48
Control: 23.1, SD 2.2
Intervention: 39.4, SD 2.1

Control normal: 1 + ((23.1-0)(10-1))/(48-0) = 5.33125
Control SD normal: (1 + (((2.2^2)-0)(10-1))/(48-0))^0.5 = 1.381

Intervention normal: 1 +((39.4-0)(10-1))/(48-0) = 8.3875
Intervention SD normal: (1 + (((2.1^2)-0)(10-1))/(48-0))^0.5 = 1.352

Nilsson C, et al.[17]:

Scale is 5-25, A = 5 B = 25
Control: 14.3, 95% CI: 11.9-16.6
Control n = 11
Intervention: 14.0, 95% CI: 11.9-16.1
Intervention n = 12

Control normal: 1 + ((14.3-5)(10-1))/(25-5) = 5.185
Control SD:

From upper limit: SD = ((11^0.5)(16.6-14.3))/1.96 = 3.892
From lower limit: SD = ((11^0.5)(14.3-11.9))/1.96 = 4.061
Thus, choosing SD from lower limit, SD = 4.061

Control SD normal: (1 + (((4.061^2)-5)(10-1))/(25-5))^0.5 = 2.484

Intervention normal: 1 + ((14.0-5)(10-1))/(25-5) = 5.05
Intervention SD:

From upper limit: SD = ((12^0.5)(16.1-14))/1.96 = 3.712
From lower limit: SD = ((12^0.5)(14-11.9))/1.96 = 3.712
Thus, as SD is the same from both limits, SD = 3.712

Intervention SD normal: (1 + (((3.712^2)-5)(10-1))/(25-5))^0.5 = 2.225

Waterman BR, et al.[57]:

Scale is 8-38, A = 8 B = 38
Control: 21.3, SD 1.5
Intervention: 22.5, SD 1.5

Control normal: 1 + ((21.3-8)(10-1))/(38-8) = 4.99
Control SD normal: (1 + (((1.5^2)-8)(10-1))/(38-8))^0.5 = 0.851

Intervention normal: 1 + ((22.5-8)(10-1))/(38-8) = 5.35
Intervention SD normal: (1 + (((1.5^2)-8)(10-1))/(38-8))^0.5 = 0.851

Shore EM, et al.[15]:

Presents only median values.

Dunn JC, et al.[58]:

Does not present number of subjects enrolled in intervention and control groups, merely the total number of subjects enrolled. As such, it is not included in synthesis, as it cannot be weighted by the number of subjects in each group.

Scale is 8-38, A = 8 B = 38
Control: 21.2, SD 6.6
Control n = NA
Intervention: 22.5, SD 7.2
Intervention n = NA

Control normal: 1 + ((21.2-8)(10-1))/(38-8) = 4.96
Control SD normal: (1 + (((6.6^2)-8)(10-1))/(38-8))^0.5 = 3.416

Intervention normal: 1 + ((22.5-8)(10-1))/(38-8) = 5.35
Intervention SD normal: (1 + (((7.2^2)-8)(10-1))/(38-8))^0.5 = 3.762

Patel NR, et al.[16]:

Scale is 9-45, A = 9 B = 45
Control: 26.2, SD 10.1
Intervention: 29.9, SD 9.8

Control normal: 1 + ((26.2-9)(10-1))/(45-9) = 5.3
Control SD normal: (1 + (((10.1^2)-9)(10-1))/(45-9))^0.5 = 4.925

Intervention normal: 1 + ((29.9-9)(10-1))/(45-9) = 6.225
Intervention SD normal: (1 + (((9.8^2)-9)(10-1))/(45-9))^0.5 = 4.771

Peltan ID, et al.[29]:

Scale is 1-5, A = 1 B = 5
Control: 2.9, SD 1.1
Intervention: 3.1, SD 1.1

Control normal: 1 + ((2.9-1)(10-1))/(5-1) = 5.275
Control SD normal: (1 + (((1.1^2)-1)(10-1))/(5-1))^0.5 = 1.213

Intervention normal: 1 + ((3.1-1)(10-1))/(5-1) = 5.725
Intervention SD normal: (1 + (((1.1^2)-1)(10-1))/(5-1))^0.5 = 1.213

Carlsen CG, et al.[59]:

Scale is 8-40, A = 8 B = 40
Control: 21.7, 95% CI: 17.3-26.1
Control n = 7
Intervention: 26.9 95% CI: 23.4-30.4
Intervention n = 9

Control normal: 1 + ((21.7-8)(10-1))/(40-8) = 4.853125
Control SD:

From upper limit: SD = ((7^0.5)(26.1-21.7))/1.96 = 5.9394
From lower limit: SD = ((7^0.5)(21.7-17.3))/1.96 = 5.9394
Thus, as SD is the same from both limits, SD = 5.9394

Control SD normal: (1 + (((5.9394^2)-8)(10-1))/(40-8))^0.5 = 2.945

Intervention normal: 1 + ((26.9-8)(10-1))/(40-8) = 6.315652
Intervention SD:

From upper limit: SD = ((9^0.5)(30.4-26.9))/1.96 = 5.357
From lower limit: SD = ((9^0.5)(26.9-23.4))/1.96 = 5.357
Thus, as SD is the same from both limits, SD = 5.357

Intervention SD normal: (1 + (((5.357^2)-8)(10-1))/(40-8))^0.5 = 2.612

Zendejas B, et al.[14]:

Scale is 6-30, A = 6 B = 30
Control: 18.3, SD 3.8
Intervention: 21.9, SD 2.7

Control normal: 1 + ((18.3-6)(10-1))/(30-6) = 5.6125
Control SD normal: (1 + (((3.8^2)-6)(10-1))/(30-6))^0.5 = 2.041

Intervention normal: 1 + ((21.9-6)(10-1))/(30-6) = 6.9625
Intervention SD normal: (1 + (((2.7^2)-6)(10-1))/(30-6))^0.5 = 1.218

Haycock A, et al.[61]:

Presents only median values

Final table of results

	Intervention			Control				Std. Mean Difference		
Study	Mean	SD	n	Mean	SD	n	Weight	Value	95% CI lower limit	95% CI upper limit
Maertens H, et al.[41]	8.39	1.35	9	5.33	1.38	10	8.6%	2.14	0.96	3.32
Nilsson C, et al.[17]	5.05	2.23	11	5.19	2.48	12	13.9%	-0.06	-0.88	0.76
Waterman BR, et al.[57]	5.35	0.85	12	4.99	0.85	10	13.4%	0.41	-0.44	1.26
Patel NR, et al.[16]	6.23	4.77	11	5.3	4.93	11	13.6%	0.18	-0.65	1.02
Peltan ID, et al.[29]	5.73	1.21	27	5.28	1.21	24	20.2%	0.37	-0.19	0.92
Carlsen CG, et al.[59]	6.32	2.6	9	4.85	2.95	7	10.8%	0.50	-0.50	1.51
Zendejas B, et al.[14]	6.96	1.22	26	5.61	2.04	24	19.6%	0.80	0.22	1.38

Expressing intervention procedure time as a fraction of the control intervention time

For all papers, control time, control time SD, intervention time and intervention time SD were divided by the control time. Thus the intervention time is expressed as a fraction of the control time.

Standard deviation, if 95% CI is given instead, calculated using:

Lower limit = mean - 1.96(SD/(n^0.5))

Upper limit = mean + 1.96(SD/(n^0.5))

Where n = number of participants in measured group

Rearranged

SD = ((n^0.5)(mean - lower limit))/1.96
SD = ((n^0.5)(upper limit - mean))/1.96

Then choosing whichever of the above give the largest value of SD

Maertens H, et al.[41]:

Control time (min): 42.6, SD 7.5
Intervention time (min): 51.3, SD 7.1

Control time fraction: 42.6/42.6 = 1
Control time SD: 7.5/42.6 = 0.176

Intervention time fraction: 51.3/42.6 = 1.204
Intervention time SD: 7.1/42.6 = 0.167

Desender L, et al.[38]:

Control time mean (min): 54.6, 95% CI: 48.4–61.6
Control n = 50
Intervention time mean (min): 52.1, 95% CI: 46.2–58.8
Intervention n = 50

Control SD

From upper limit: SD = ((50^0.5)(61.6-54.6))/1.96 = 25.2538
From lower limit: SD = ((50^0.5)(54.6-48.4))/1.96 = 22.3677
Thus, as SD from the upper limit is larger, SD = 25.2538

Intervention SD

From upper limit: SD = ((50^0.5)(58.8-52.1))/1.96 = 24.1715
From lower limit: SD = ((50^0.5)(52.1-46.2))/1.96 = 21.2854
Thus, as SD from upper limit is larger, SD = 24.1715

Control time fraction: 54.6/54.6 = 1
Control time SD: 25.2538/54.6 = 0.4625

Intervention time fraction: 52.1/54.6 = 0.9542
Intervention time SD: 24.1715/54.6 = 0.4427

Waterman BR, et al.[57]:

Control time (sec): 232; SD 106
Intervention time (sec): 205.9; SD 105.2

Control time fraction: 232/232 = 1
Control time SD: 106/232 = 0.4569

Intervention time fraction: 205.9/232 = 0.8875
Intervention time SD: 105.2/232 = 0.4534

Shore EM, et al.[15]:

Presents only median values.

Dunn JC, et al.[58]:

Does not present number of subjects enrolled in intervention and control groups, merely the total number of subjects enrolled. As such, it is not included in synthesis, as it cannot be weighted by the number of subjects in each group.

Carlsen CG, et al.[59]:

Control time (min): 61.3; 95% CI 39.3-83.3
Control n = 7
Intervention time (min): 40.1; 95% CI 33.0-47.2
Intervention n = 9

Control SD:

From upper limit: SD = ((7^0.5)(83.3-61.3))/1.96 = 29.6972
From lower limit: SD = ((7^0.5)(61.3-39.3))/1.96 = 29.6972
Thus, as SD is the same from both limits, SD = 29.6972

Intervention SD:

From upper limit: SD = ((9^0.5)(47.2-40.1))/1.96 = 10.867
From lower limit: SD = ((9^0.5)(40.1-33))/1.96 = 10.867

Thus, as SD is the same from both limits, SD = 10.867

Control time fraction: 61.3/61.3 = 1
Control time SD: 29.6972/61.3 = 0.4845

Intervention time fraction: 40.1/61.3 = 0.6542
Intervention time SD: 10.867/61.3 = 0.1773

Zendejas B, et al.[14]:

Control time raw (min): 37.4; SD 8.3
Intervention time raw (min): 30.9; SD 7.3

Control time fraction: 37.4/37.4 = 1
Control time SD: 8.3/37.4 = 0.222

Intervention time fraction: 30.9/37.4 = 0.8262
Intervention time SD: 7.3/37.4 = 0.1952

Haycock A, et al.[61]:

Presents only median values

Final table of results

	Intervention			Control				Std. Mean Difference		
Study	Mean	SD	n	Mean	SD	n	Weight	Value	95% CI lower limit	95% CI upper limit
Maertens H, et al.[41]	1.204	0.167	9	1	0.176	10	16.20%	1.13	0.15	2.12
Desender L, et al.[38]	0.9542	0.4427	50	1	0.4625	50	26.70%	-0.1	-0.49	0.29
Waterman BR, et al.[57]	0.8875	0.4534	12	1	0.4569	10	18.60%	-0.24	-1.08	0.6
Carlsen CG, et al.[59]	0.6542	0.1773	9	1	0.4845	7	15.20%	-0.95	-2.01	0.11
Zendejas B, et al.[14]	0.8262	0.1952	26	1	0.222	24	23.40%	-0.82	-1.4	-0.24
