# Supplementary material for: The impact of surgical simulation on patient outcomes: a systematic review and meta-analysis
Source: Neurosurg Rev. 2020 May 13;44(2):843–54. doi: 10.1007/s10143-020-01314-2 (PMC8035110; doi:10.1007/s10143-020-01314-2)
Supplement: Supplementary file 2 — . PDF. Screening. Results of the initial screening of 119 articles assessed for eligibility. (PDF 80 kb) [file 10143_2020_1314_MOESM2_ESM.pdf]

Supplemental File 2. PDF. Screening

Paper Number	Authors	Citation	Cause for exclusion	Excluded in pass number	Notes
1	Mohan D et.al.	Mohan D, Rosengart MR, Fischhoff B, et al. Testing a videogame intervention to recalibrate physician heuristics in trauma triage: study protocol for a randomized controlled trial. BMC Emerg Med. 2016;16(1):44.	Does not simulate manual skills or surgery	1	Triage of trauma patients
2	Overschmidt B et. al.	Overschmidt B, Qureshi AA, Parikh RP, Yan Y, Tenenbaum MM, Myckatyn TM. A Prospective Evaluation of Three-Dimensional Image Simulation: Patient-Reported Outcomes and Mammometrics in Primary Breast Augmentation. Plast Reconstr Surg. 2018;142(2):133e-144e.	Does not simulate manual skills or surgery	1	3D simulation before breast plastic surgery
3	Hohnloser SH et. al.	Hohnloser SH, Camm J, Cappato R, et al. Uninterrupted administration of edoxaban vs vitamin K antagonists in patients undergoing atrial fibrillation catheter ablation: Rationale and design of the ELIMINATE-AF study. Clin Cardiol. 2018;41(4):440-449.	Does not simulate manual skills or surgery	1	Study design of RCT testing non vit k vs vit k anticoagulants in AF patients post op
4	Wooster M et. al.	Wooster M, Doyle A, Hislop S, et al. REHEARSAL Using Patient-Specific Simulation to Improve Endovascular Efficiency. Vasc Endovascular Surg. 2018;52(3):169-172.	No		Carotid endovascular stenting, patients anatomy uploaded to sim and practised on before actual operation
5	Smith AF et. al.	Smith AF, Hall PS, Hulme CT, et al. Cost-effectiveness analysis of PET-CT-guided management for locally advanced head and neck cancer. Eur J Cancer. 2017;85:6-14.	Does not simulate manual skills or surgery	1	Cost effectiveness analysis of PET-CT in head and neck cancer
6	Branzetti JB et. al.	Branzetti JB, Adedipe AA, Gittinger MJ, et al. Randomised controlled trial to assess the effect of a Just-in-Time training on procedural performance: a proof-of-concept study to address procedural skill decay. BMJ Qual Saf. 2017;26(11):881-891.	Only in simulation/no patients	1	Pacemaker placement in manequin
7	Bengtsson M et. al.	Bengtsson M, Wall G, Greiff L, Rasmusson L. Treatment outcome in orthognathic surgery-A prospective randomized blinded case-controlled comparison of planning accuracy in computer-assisted two- and three-dimensional planning techniques (part II). J Craniomaxillofac Surg. 2017;45(9):1419-1424.	Does not simulate manual skills or surgery	1	2D vs 3D prediction of outcome after cranio-maxillo-facio surgery
8	Maertens H et. al.	Maertens H, Aggarwal R, Moreels N, Vermassen F, Van Herzeele I. A proficiency based stepwise endovascular curricular training (PROSPECT) program enhances operative performance in real life: A randomised controlled trial. Eur J Vasc Endovasc Surg. 2017;54(3):387-396.	No		Endovascular interventions in lower extremity
9	Rieger B et. al.	Rieger B, Jiang H, Reinshagen C, et al. Effects of preoperative simulation on minimally invasive hybrid lumbar interbody fusion. World Neurosurg. 2017;106:578-588.	Does not simulate manual skills or surgery	1	Lumbar inter-body fusion, patients specific anatomy constructed pre-op
10	Eshghpour M et. al.	Eshghpour M, Shaban B, Ahrari F, Erfanian M, Shadkam E. Is Low-Level Laser Therapy Effective for Treatment of Neurosensory Deficits Arising From Sagittal Split Ramus Osteotomy? J Oral Maxillofac Surg. 2017;75(10):2085-2090.	Does not simulate manual skills or surgery	1	Laser treatment after cranio-maxillo-facio surgery
11	Zevin B et. al.	Zevin B, Dedy NJ, Bonrath EM, Grantcharov TP. Comprehensive simulation-enhanced training curriculum for an advanced minimally invasive procedure: a randomized controlled trial. Surg Obes Relat Dis. 2017;13(5):815-824.	No		Bariatric surgery, practice and assessement on pigs, intervention group rated on live patient after trial
12	Zonnebeld N et. al.	Zonnebeld N, Huberts W, van Loon MM, Delhaas T, Tordoir JHM. Preoperative computer simulation for planning of vascular access surgery in hemodialysis patients. J Vasc Access. 2017;18(Suppl. 1):118-124.	Does not simulate manual skills or surgery	1	Pre-operative assessment of arterio-venous fistula construction
13	Gerretsen P et. al.	Gerretsen P, Pothier DD, Falls C, et al. Vestibular stimulation improves insight into illness in schizophrenia spectrum disorders. Psychiatry Res. 2017;251:333-341.	Does not simulate manual skills or surgery	1	Caloric vestibular simulation in patients with schizophrenia
14	Desender L et. al.	Desender L, Van Herzeele I, Lachat M, et al. A Multicentre Trial of Patient specific Rehearsal Prior to EVAR: Impact on Procedural Planning and Team Performance. Eur J Vasc Endovasc Surg. 2017;53(3):354-361.	No		Aneurism repair, half of patients anatomy uploaded to sim and practised on before actual operation
15	Bekelis K et. al.	Bekelis K, Calnan D, Simmons N, MacKenzie TA, Kakoulides G. Effect of an immersive preoperative virtual reality experience on patient reported outcomes: A randomized controlled trial. Ann Surg. 2017;265(6):1068-1073.	Does not simulate manual skills or surgery	1	Simulation to patients to prevent perioperative stress
16	Mohan D et.al.	Mohan D, Fischhoff B, Angus DC, et al. Serious games may improve physician heuristics in trauma triage. Proc Natl Acad Sci USA. 2018;115(37):9204-9209.	Does not simulate manual skills or surgery	1	Triage of trauma patients
17	Amorfini L et. al.	Amorfini L, Migliorati M, Drago S, Silvestrini-Biavati A. Immediately Loaded Implants in Rehabilitation of the Maxilla: A Two-Year Randomized Clinical Trial of Guided Surgery versus Standard Procedure. Clin Implant Dent Relat Res. 2017;19(2):280-295.	Does not simulate manual skills or surgery	1	Patient anatomy re-created to create better tooth implants
18	You W et. al.	You W, Liu LJ, Chen HX, et al. Application of 3D printing technology on the treatment of complex proximal humeral fractures (Neer3-part and 4-part) in old people. Orthop Traumatol Surg Res. 2016;102(7):897-903.	Does not simulate manual skills or surgery	1	3D printing of humeral fractures
19	Nilsson C et. al.	Nilsson C, Sorensen JL, Konge L, et al. Simulation-based camera navigation training in laparoscopy-a randomized trial. Surg Endosc. 2017;31(5):2131-2139.	No		Simulation of endoscopic camera navigation. simulation test, then transfer test in actual OR navigating camera in a cholecystectomy
20	Sampaio-Filho H et. al.	Sampaio-Filho H, Sotto-Ramos J, Pinto EH, et al. Evaluation of low-level laser at auriculotherapy points to reduce postoperative pain in inferior third molar surgery: study protocol for a randomized controlled trial. Trials. 2016;17(1):432.	Does not simulate manual skills or surgery	1	Study design of RCT testing laser therapy after 3. molar surgery
21	Broekema TH et. al.	Broekema TH, Talsma AK, Wevers KP, Pierie J-PEN. Laparoscopy instructional videos: the effect of preoperative compared with intraoperative use on learning curves. J Surg Educ. 2017;74(1):91-99.	Only in simulation/no patients	1	Effect of instructional videos on simulation proficiency
22	Skelton T et. al.	Skelton T, Nshimyumuremyi I, Mukwesi C, Whynot S, Zolpys L, Livingston P. Low-Cost Simulation to Teach Anesthetists' Non-Technical Skills in Rwanda. Anesth Analg. 2016;123(2):474-480.	Does not simulate manual skills or surgery	1	Anesthesia administration
23	Tang Q et. al.	Tang Q, Sun Z, Zhang N, et al. Cost-Effectiveness of Bariatric Surgery for Type 2 Diabetes Mellitus: A Randomized Controlled Trial in China. Medicine (Baltimore). 2016;95(20):e3522.	Does not simulate manual skills or surgery	1	Cost effectiveness analysis of bariatric surgery for T2DM patients
24	Waterman BR et. al.	Waterman BR, Martin KD, Cameron KL, Owens BD, Belmont PJ. Simulation training improves surgical proficiency and safety during diagnostic shoulder arthroscopy performed by residents. Orthopedics. 2016;39(3):e479-85.	No		Diagnostic shoulder arthroscopy
25	Shore EM et. al.	Shore EM, Grantcharov TP, Husslein H, et al. Validating a standardized laparoscopy curriculum for gynecology residents: a randomized controlled trial. Am J Obstet Gynecol. 2016;215(2):204.e1-204.e11.	No		Salpingectomy and intracorporeal knot tying, assessment in OR
26	Neubauer AS et. al.	Neubauer AS, Reznicek L, Minartz C, Ziemssen F. [Economic Short-Term Cost Model for Stereotactic Radiotherapy of Neovascular AMD]. Klin Monbl Augenheilkd. 2016;233(8):951-957.	Does not simulate manual skills or surgery	1	Cost effectiveness of stereotactic radiation therapy on AMD
27	Eshghpour M et. al.	Eshghpour M, Ahrari F, Takallu M. Is Low-Level Laser Therapy Effective in the Management of Pain and Swelling After Mandibular Third Molar Surgery? J Oral Maxillofac Surg. 2016;74(7):1322.e1-8.	Does not simulate manual skills or surgery	1	Laser treatment after cranio-maxillo-facio surgery
28	Kim AH et. al.	Kim AH, Kendrick DE, Moorehead PA, et al. Endovascular aneurysm repair simulation can lead to decreased fluoroscopy time and accurately delineate the proximal seal zone. J Vasc Surg. 2016;64(1):251-258.	Only in simulation/no patients	1	Aortic aneurism stenting on simulator
29	Engblom H et. al.	Engblom H, Heiberg E, Erlinge D, et al. Sample size in clinical cardioprotection trials using myocardial salvage index, infarct size, or biochemical markers as endpoint. J Am Heart Assoc. 2016;5(3):e002708.	Does not simulate manual skills or surgery	1	Determining sample size needed for cardioprotection trials
30	Steinemann S et. al.	Steinemann S, Bhatt A, Suares G, et al. Trauma team discord and the role of briefing. J Trauma Acute Care Surg. 2016;81(1):184-189.	Does not simulate manual skills or surgery	1	Effect of briefing on trauma team proficiency
31	Beckmann M et. al.	Beckmann M, Merollini K, Kumar S, Flenady V. Induction of labor using prostaglandin vaginal gel: cost analysis comparing early amniotomy with repeat prostaglandin gel. Eur J Obstet Gynecol Reprod Biol. 2016;199:96-101.	Does not simulate manual skills or surgery	1	Cost analysis of modes of induction of labour
32	Radcliff K et. al.	Radcliff K, Lerner J, Yang C, Bernard T, Zigler JE. Seven-year cost-effectiveness of ProDisc-C total disc replacement: results from investigational device exemption and post-approval studies. J Neurosurg Spine. 2016;24(5):760-768.	Does not simulate manual skills or surgery	1	Cost analysis of modes of cervical spine surgery
33	Li Y et. al.	Li Y, Zhao Y, Zhang J, et al. Low-Cost Interactive Image-Based Virtual Endoscopy for the Diagnosis and Surgical Planning of Suprasellar Arachnoid Cysts. World Neurosurg. 2016;88:76-82.	Does not simulate manual skills or surgery	1	Recreation of cysts in computer software in order to plan surgery
34	Wolf E et. al.	Wolf E, Utech M, Stehle P, et al. Oral High-Dose Vitamin D Dissolved in Oil Raised Serum 25-Hydroxy-Vitamin D to Physiological Levels in Obese Patients After Sleeve Gastrectomy-A Double-Blind, Randomized, and Placebo-Controlled Trial. Obes Surg. 2016;26(8):1821-1829.	Does not simulate manual skills or surgery	1	Vit D in patients post bariatric surgery
35	Johnston MJ et. al.	Johnston MJ, Arora S, Pucher PH, et al. Improving Escalation of Care: A Double-blinded Randomized Controlled Trial. Ann Surg. 2016;263(3):421-426.	Only in simulation/no patients	1	Escalation of care in surgery
36	Patel NR et. al.	Patel NR, Makai GE, Sloan NL, Della Badia CR. Traditional versus simulation resident surgical laparoscopic salpingectomy training: A randomized controlled trial. J Minim Invasive Gynecol. 2016;23(3):372-377.	No		Salpingectomy, porcine simulation model, human pre and post test
37	Osnabrugge RL et. al.	Osnabrugge RL, Magnuson EA, Serruys PW, et al. Cost-effectiveness of percutaneous coronary intervention versus bypass surgery from a Dutch perspective. Heart. 2015;101(24):1980-1988.	Does not simulate manual skills or surgery	1	Cost effectiveness of PCI vs bypass surgery
38	Kiely DJ et. al.	Kiely DJ, Gotlieb WH, Lau S, et al. Virtual reality robotic surgery simulation curriculum to teach robotic suturing: a randomized controlled trial. J Robot Surg. 2015;9(3):179-186.	Only in simulation/no patients	1	Da Vinci suturing of vaginal cuff model
39	Wong CK et. al.	Wong CKH, Lam CLK, Wan YF, Fong DYT. Cost-effectiveness simulation and analysis of colorectal cancer screening in Hong Kong Chinese population: comparison amongst colonoscopy, guaiac and immunologic fecal occult blood testing. BMC Cancer. 2015;15:705.	Does not simulate manual skills or surgery	1	Cost effectiveness of different modalities for screening of colorectal cancer
40	Waha JE et. al.	Waha JE, Goswami N, Schlagenhauf A, et al. Effects of exercise and nutrition on the coagulation system during bedrest immobilization. Medicine (Baltimore). 2015;94(38):e1555.	Does not simulate manual skills or surgery	1	Effect of nutrition + exercise on coagulation in patients needing bedrest
41	Shavit I et. al.	Shavit I, Levit B, Basat NB, Lait D, Somri M, Gaitini L. Establishing a definitive airway in the trauma patient by novice intubators: A randomised crossover simulation study. Injury. 2015;46(11):2108-2112.	Only in simulation/no patients	1	Intubating blind vs with larygoscope
42	Walker DM et. al.	Walker DM, Cohen SR, Fritz J, et al. Impact Evaluation of PRONTO Mexico: A Simulation-Based Program in Obstetric and Neonatal Emergencies and Team Training. Simul Healthc. 2016;11(1):1-9.	Does not simulate manual skills or surgery	1	Hospitals recieved training in managing obstetric hemorrhage, neonatal recussitation, shoulder dystocia, pre-eclampsia and eclampsia
43	Dunn JC et. al.	Dunn JC, Belmont PJ, Lanzi J, et al. Arthroscopic shoulder surgical simulation training curriculum: transfer reliability and maintenance of skill over time. J Surg Educ. 2015;72(6):1118-1123.	No		Arthroscopy og shoulder joint. In vivo test, then simulation, then another in vivo test. Then 1 year later new in vivo test to look at skill decay
44	Makhdom AM et. al.	Makhdom AM, Almaawi A, Tanzer D, Tanzer M. Does warming up improve surgical outcome in total hip arthroplasty? Eur J Orthop Surg Traumatol. 2015;25(8):1265-1269.	Does not simulate manual skills or surgery	1	1. vs 2. surgery of the day, which has a better outcome?
45	Kjellberg G et. al.	Kjellberg G, Sartipy U, van der Linden J, Nissborg E, Lindvall G. An Adjusted Calculation Model Allows for Reduced Protamine Doses without Increasing Blood Loss in Cardiac Surgery. Thorac Cardiovasc Surg. 2016;64(6):487-493.	Does not simulate manual skills or surgery	1	Heparin and protamine doses after cardiac surgery
46	Peltan ID et. al.	Peltan ID, Shiga T, Gordon JA, Currier PF. Simulation improves procedural protocol adherence during central venous catheter placement: A randomized controlled trial. Simul Healthc. 2015;10(5):270-276.	No		Central venous catheter placement
47	You-Ten KE et. al.	You-Ten KE, Wong C, Arzola C, et al. Role of contextualizing a crisis scenario on the performance of a cricothyrotomy procedural task. Can J Anaesth. 2015;62(10):1104-1113.	Only in simulation/no patients	1	Cricothyrotomy, kit then porcine model
48	Di Marco AN et. al.	Di Marco AN, Jeyakumar J, Pratt PJ, Yang G-Z, Darzi AW. Evaluating a novel 3D stereoscopic visual display for transanal endoscopic surgery: A randomized controlled crossover study. Ann Surg. 2016;263(1):36-42.	Only in simulation/no patients	1	Assessing different viewing modalities in trans-anal endoscopy
49	Grover SC et. al.	Grover SC, Garg A, Scaffidi MA, et al. Impact of a simulation training curriculum on technical and nontechnical skills in colonoscopy: a randomized trial. Gastrointest Endosc. 2015;82(6):1072-1079.	No		Colonoscopy, simulation pre-test, training, post-test then in-vivo post-post-test
50	Aberle SJ et. al.	Aberle SJ, Sandefur BJ, Sunga KL, et al. Intubation Efficiency and Perceived Ease of Use of Video Laryngoscopy vs Direct Laryngoscopy While Wearing HazMat PPE: A Preliminary High-fidelity Mannequin Study. Prehosp Disaster Med. 2015;30(3):259-263.	Only in simulation/no patients	1	Video vs direct laryngoscopy while wearing hazmat suits
51	Størset E et. al.	Størset E, Åsberg A, Skauby M, et al. Improved Tacrolimus Target Concentration Achievement Using Computerized Dosing in Renal Transplant Recipients--A Prospective, Randomized Study. Transplantation. 2015;99(10):2158-2166.	Does not simulate manual skills or surgery	1	Tacrolimus dose after kidney transplant
52	Martin KD et. al.	Martin KD, Patterson D, Phisitkul P, Cameron KL, Femino J, Amendola A. Ankle arthroscopy simulation improves basic skills, anatomic recognition, and proficiency during diagnostic examination of residents in training. Foot Ankle Int. 2015;36(7):827-835.	Only in simulation/no patients	1	Ankle arthroscopy
53	Carlsen CG et. al.	Carlsen CG, Lindorff-Larsen K, Funch-Jensen P, Lund L, Konge L, Charles P. Module based training improves and sustains surgical skills: a randomised controlled trial. Hernia. 2015;19(5):755-763.	No		Inguinal hernia repair. Mannequin and pig simution then in-vivo testing.
54	Rajan SS et. al.	Rajan SS, Sharma SC, Kumar N, et al. Clinical and cosmetic results of breast boost radiotherapy in early breast cancer: a randomized study between electron and photon. J Cancer Res Ther. 2014;10(4):889-895.	Does not simulate manual skills or surgery	1	Radiation treatment regimen for BC
55	Maytin M et. al.	Maytin M, Daily TP, Carillo RG. Virtual reality lead extraction as a method for training new physicians: a pilot study. Pacing Clin Electrophysiol. 2015;38(3):319-325.	Does not simulate manual skills or surgery	1	Heart lead extraction
56	Koch AD et. al.	Koch AD, Ekkelenkamp VE, Haringsma J, Schoon EJ, de Man RA, Kuipers EJ. Simulated colonoscopy training leads to improved performance during patient-based assessment. Gastrointest Endosc. 2015;81(3):630-636.	No		Colonoscopy, both groups recieved VR training but different amounts
57	Kinoshita H et. al.	Kinoshita H, Nakagawa K, Usui Y, et al. High-definition resolution three-dimensional imaging systems in laparoscopic radical prostatectomy: randomized comparative study with high-definition resolution two-dimensional systems. Surg Endosc. 2015;29(8):2203-2209.	Does not simulate manual skills or surgery	1	2D vs 3D imagining during prostatectomy
58	Punke MA et. al.	Punke MA, Goepfert MS, Kluge S, Reichenspurner H, Goetz AE, Reuter DA. Perioperative glycemic control with a computerized algorithm versus conventional glycemic control in cardiac surgical patients undergoing cardiopulmonary bypass with blood cardioplegia. J Cardiothorac Vasc Anesth. 2014;28(5):1273-1277.	Does not simulate manual skills or surgery	1	control of blood glucose levels in bypass patients
59	Sorensen LS et. al.	Sorensen LS, Thorlacius-Ussing O, Rasmussen HH, et al. Effects of perioperative supplementation with omega-3 fatty acids on leukotriene B_4 and leukotriene B_5 production by stimulated neutrophils in patients with colorectal cancer: a randomized, placebo-controlled intervention trial. Nutrients. 2014;6(10):4043-4057.	Does not simulate manual skills or surgery	1	Omega 3 supplementation to patients with colorectal cancer
60	Hwang M et. al.	Hwang M, Kwon S-S, Wi J, et al. Virtual ablation for atrial fibrillation in personalized in-silico three-dimensional left atrial modeling: comparison with clinical catheter ablation. Prog Biophys Mol Biol. 2014;116(1):40-47.	Only in simulation/no patients	1	Retroactive recreation of atriae with AF, then simulating different treatment strategies
61	Moosavi H et. al.	Moosavi H, Maleknejad F, Sharifi M, Ahrari F. A randomized clinical trial of the effect of low-level laser therapy before composite placement on postoperative sensitivity in class V restorations. Lasers Med Sci. 2015;30(4):1245-1249.	Does not simulate manual skills or surgery	1	Laser treatment on teeth
62	Boggs DH et. al.	Boggs DH, Feigenberg S, Walter R, et al. Stereotactic radiotherapy using tomotherapy for early-stage non-small cell lung carcinoma: analysis of intrafraction tumour motion. J Med Imaging Radiat Oncol. 2014;58(6):706-713.	Does not simulate manual skills or surgery	1	Radiation treatment of lung cancers
63	Nickel F et. al.	Nickel F, Jede F, Minassian A, et al. One or two trainees per workplace in a structured multimodality training curriculum for laparoscopic surgery? Study protocol for a randomized controlled trial - DRKS00004675. Trials. 2014;15:137.	Only in simulation/no patients	1	Laparascopic cholecystectomy
64	Castells A et. al.	Castells A, Quintero E, Álvarez C, et al. Rate of detection of advanced neoplasms in proximal colon by simulated sigmoidoscopy vs fecal immunochemical tests. Clin Gastroenterol Hepatol. 2014;12(10):1708-16.e4.	Does not simulate manual skills or surgery	1	Screening for colorectal cancer
65	Fang CH et. al.	Fang C, Kong D, Wang X, et al. Three-dimensional reconstruction of the peripancreatic vascular system based on computed tomographic angiography images and its clinical application in the surgical management of pancreatic tumors. Pancreas. 2014;43(3):389-395.	Does not simulate manual skills or surgery	1	3D reconstruction of arteries in treatment of pancreatic cancer
66	De Riu G et. al.	De Riu G, Meloni SM, Baj A, Corda A, Soma D, Tullio A. Computer-assisted orthognathic surgery for correction of facial asymmetry: results of a randomised controlled clinical trial. Br J Oral Maxillofac Surg. 2014;52(3):251-257.	Does not simulate manual skills or surgery	1	Computer assisted facial surgery
67	Schebesta et. al.	Schebesta K, Karanovic G, Krafft P, Rössler B, Kimberger O. Distance from the glottis to the grille: the LMA Unique, Air-Q and CobraPLA as intubation conduits: a randomised trial. Eur J Anaesthesiol. 2014;31(3):159-165.	Does not simulate manual skills or surgery	1	Anatomical distances in different supra-glottic devices
68	Chiang MH et. al.	Chiang M-H, Wu S-C, You C-H, et al. Target-controlled infusion vs. manually controlled infusion of propofol with alfentanil for bidirectional endoscopy: a randomized controlled trial. Endoscopy. 2013;45(11):907-914.	Does not simulate manual skills or surgery	1	Types of anesthesia for endoscopy
69	Jepsen CH et. al.	Jepsen CH, Gätke MR, Thøgersen B, et al. Tracheal intubation with a flexible fibreoptic scope or the McGrath videolaryngoscope in simulated difficult airway scenarios: a randomised controlled manikin study. Eur J Anaesthesiol. 2014;31(3):131-136.	Only in simulation/no patients	1	Types of intubation on a manequin
70	Hisashige A et. al.	Hisashige A, Sasako M, Nakajima T. Cost-effectiveness of adjuvant chemotherapy for curatively resected gastric cancer with S-1. BMC Cancer. 2013;13:443.	Does not simulate manual skills or surgery	1	Cost effectiveness of chemotherapy on gastric cancer post op
71	Bambakidis NC et. al.	Bambakidis NC, Selman WR, Sloan AE. Surgical rehearsal platform: potential uses in microsurgery. Neurosurgery. 2013;73 Suppl 1:122-126.	Does not simulate manual skills or surgery	1	Description of the Selman Surgical Rehearsal Platform
72	Berry JL et. al.	Berry JL, Dandapani SV, Stevanovic M, et al. Outcomes of choroidal melanomas treated with eye physics: a 20-year review. JAMA Ophthalmol. 2013;131(11):1435-1442.	Does not simulate manual skills or surgery	1	Treatment of choroidal melanomas
73	Sørensen JL et. al.	Sørensen JL, Van der Vleuten C, Lindschou J, et al. "In situ simulation" versus "off site simulation" in obstetric emergencies and their effect on knowledge, safety attitudes, team performance, stress, and motivation: study protocol for a randomized controlled trial. Trials. 2013;14:220.	Does not simulate manual skills or surgery	1	ISS vs OSS RCT study protocol
74	Hung RK et. al.	Hung RKY, Lewinsohn A, Jovaisa T, Wijayatilake DS, Sherren PB. Does the use of a bougie reduce the force of laryngoscopy in a difficult airway with manual in-line stabilisation?: a randomised crossover simulation study. Eur J Anaesthesiol. 2013;30(9):563-566.	Only in simulation/no patients	1	Neck support when intubating
75	Logan H et. al.	Logan H, Wolfaardt J, Boulanger P, Hodgetts B, Seikaly H. Exploratory benchtop study evaluating the use of surgical design and simulation in fibula free flap mandibular reconstruction. J Otolaryngol Head Neck Surg. 2013;42:42.	Only in simulation/no patients	1	Reconstruction of 3D printed mandibles
76	Jain N et. al.	Jain N, Anderson MJ, Patel P, et al. Melanoma simulation model: promoting opportunistic screening and patient counseling. JAMA Dermatol. 2013;149(6):710-716.	Does not simulate manual skills or surgery	1	Identification of melanomas
77	Phillips CL et. al.	Phillips CL, Grunstein RR, Darendeliler MA, et al. Health outcomes of continuous positive airway pressure versus oral appliance treatment for obstructive sleep apnea: a randomized controlled trial. Am J Respir Crit Care Med. 2013;187(8):879-887.	Does not simulate manual skills or surgery	1	CPAP vs MAD in obstructive sleep apnea
78	Shaw TJ et. al.	Shaw TJ, Pernar LI, Peyre SE, et al. Impact of online education on intern behaviour around joint commission national patient safety goals: a randomised trial. BMJ Qual Saf. 2012;21(10):819-825.	Only in simulation/no patients	1	Patient safety
79	McIntyre CC et. al.	McIntyre CC, Richardson SJ, Frankemolle AM, Varga G, Noecker AM, Alberts JL. Improving postural stability via computational modeling approach to deep brain stimulation programming. Conf Proc IEEE Eng Med Biol Soc. 2011;2011:675-676.	Does not simulate manual skills or surgery	1	Computer modeling of patient to determine where to apply current in deep brainstem simulation
80	Edrich T et. al.	Edrich T, Frendl G, Rawn JD, Paschalidis Y. Modeling the effects of bivalirudin in cardiac surgical patients. Conf Proc IEEE Eng Med Biol Soc. 2011;2011:120-123.	Does not simulate manual skills or surgery	1	Bivalirudin in heparin-induced thrombocytopenia
81	Wetsch WA et. al.	Wetsch WA, Carlitscheck M, Spelten O, et al. Success rates and endotracheal tube insertion times of experienced emergency physicians using five video laryngoscopes: a randomised trial in a simulated trapped car accident victim. Eur J Anaesthesiol. 2011;28(12):849-858.	Only in simulation/no patients	1	Different laryngoscopes in intubation
82	Benoit E et. al.	Benoit E, O'Donnell TF, Iafrati MD, et al. The role of amputation as an outcome measure in cellular therapy for critical limb ischemia: implications for clinical trial design. J Transl Med. 2011;9:165.	Does not simulate manual skills or surgery	1	Factors influencing amputation
83	Zendejas B et. al.	Zendejas B, Cook DA, Bingener J, et al. Simulation-based mastery learning improves patient outcomes in laparoscopic inguinal hernia repair: a randomized controlled trial. Ann Surg. 2011;254(3):502-509; discussion 509.	No		Laparascopic inguinal hernia repair
84	Aggarwal R et. al.	Aggarwal R, Mishra A, Crochet P, Sirimanna P, Darzi A. Effect of caffeine and taurine on simulated laparoscopy performed following sleep deprivation. Br J Surg. 2011;98(11):1666-1672.	Only in simulation/no patients	1	Caffeine and taurine on sleep-deprived operators
85	Kessler DO et. al.	Kessler DO, Auerbach M, Pusic M, Tunik MG, Foltin JC. A randomized trial of simulation-based deliberate practice for infant lumbar puncture skills. Simul Healthc. 2011;6(4):197-203.	No		Lumbar puncture on infants, self reported success after simulation training
86	Dziegielewski PT et. al.	Dziegielewski PT, Zhu J, King B, et al. Three-dimensional biomodeling in complex mandibular reconstruction and surgical simulation: prospective trial. J Otolaryngol Head Neck Surg. 2011;40 Suppl 1:S70-81.	Only in simulation/no patients	1	Bending plates to repair mandibular defects
87	Yeh ML et. al.	Yeh M-L, Chung Y-C, Chen K-M, Tsou M-Y, Chen H-H. Acupoint electrical stimulation reduces acute postoperative pain in surgical patients with patient-controlled analgesia: a randomized controlled study. Altern Ther Health Med. 2010;16(6):10-18.	Does not simulate manual skills or surgery	1	Acupoint treatmen of pain in spinal surgery patients
88	Chung TN et. al	Chung TN, Kim SW, You JS, Cho YS, Chung SP, Park I. A higher chest compression rate may be necessary for metronome-guided cardiopulmonary resuscitation. Am J Emerg Med. 2012;30(1):226-230.	Does not simulate manual skills or surgery	1	Chest compression rate and depth in CPR with a metronome
89	Schalk R et. al.	Schalk R, Engel S, Meininger D, et al. Disposable laryngeal tube suction: standard insertion technique versus two modified insertion techniques for patients with a simulated difficult airway. Resuscitation. 2011;82(2):199-202.	Does not simulate manual skills or surgery	1	Necessity of jaw thrust when intubating
90	Yang X et. al.	Yang X, Hu J, Yin G, Hu J, Luo E. Computer-assisted condylar reconstruction in bilateral ankylosis of the temporomandibular joint using autogenous coronoid process. Br J Oral Maxillofac Surg. 2011;49(8):612-617.	Does not simulate manual skills or surgery	1	Pre-operative modeling before surgery of temporomandibular joint
91	Calatayud D et. al.	Calatayud D, Arora S, Aggarwal R, et al. Warm-up in a virtual reality environment improves performance in the operating room. Ann Surg. 2010;251(6):1181-1185.	No		Warm-up in simulator or not before laparoscopic cholecystectomi
92	Park CS et. al.	Park CS, Rochlen LR, Yaghmour E, et al. Acquisition of critical intraoperative event management skills in novice anesthesiology residents by using high-fidelity simulation-based training. Anesthesiology. 2010;112(1):202-211.	Only in simulation/no patients	1	Anesthesiology, critically ill patient scenarios
93	Haycock A et. al.	Haycock A, Koch AD, Familiari P, et al. Training and transfer of colonoscopy skills: a multinational, randomized, blinded, controlled trial of simulator versus bedside training. Gastrointest Endosc. 2010;71(2):298-307.	No		Simulator training in colonoscopy
94	Day T et. al.	Day T, Iles N, Griffiths P. Effect of performance feedback on tracheal suctioning knowledge and skills: randomized controlled trial. J Adv Nurs. 2009;65(7):1423-1431.	Only in simulation/no patients	1	Tracheal suctioning
95	Filipovic N et. al.	Filipovic N, Cvetkovic A, Isailovic V, Matovic Z, Rosic M, Kojic M. Computer simulation of flow and mixing at the duodenal stump after gastric resection. World J Gastroenterol. 2009;15(16):1990-1998.	Does not simulate manual skills or surgery	1	Simulation of duodenal stump after Billroth treatment in 2 cases
96	Domuracki KJ et. al.	Domuracki KJ, Moule CJ, Owen H, Kostandoff G, Plummer JL. Learning on a simulator does transfer to clinical practice. Resuscitation. 2009;80(3):346-349.	Does not simulate manual skills or surgery	1	Pressure placed on cricoid in anesthetised patients
97	Khaykin Y et. al.	Khaykin Y, Wang X, Natale A, et al. Cost comparison of ablation versus antiarrhythmic drugs as first-line therapy for atrial fibrillation: an economic evaluation of the RAAFT pilot study. J Cardiovasc Electrophysiol. 2009;20(1):7-12.	Does not simulate manual skills or surgery	1	Cost effectiveness of radiofrequency ablation vs antiarrhythmic drugs in patients with AF
98	Blønd L et. al.	Blønd L, Jensen NV, Søe Nielsen NH. Clinical consequences of different exsanguination methods in hand surgery. a double-blind randomised study. J Hand Surg Eur Vol. 2008;33(4):475-477.	Does not simulate manual skills or surgery	1	Methods of exsanguination in hand surgery
99	Crofts JF et. al.	Crofts JF, Bartlett C, Ellis D, Hunt LP, Fox R, Draycott TJ. Management of shoulder dystocia: skill retention 6 and 12 months after training. Obstet Gynecol. 2007;110(5):1069-1074.	Does not simulate manual skills or surgery	1	Delivery and shoulder dystocia
100	Suzuki N et. al.	Suzuki N, Nanda H, Angiolillo DJ, et al. Assessment of potential relationship between wall shear stress and arterial wall response after bare metal stent and sirolimus-eluting stent implantation in patients with diabetes mellitus. Int J Cardiovasc Imaging. 2008;24(4):357-364.	Does not simulate manual skills or surgery	1	DES vs BMS stents in patients with diabetes
101	Ahlberg G et. al.	Ahlberg G, Enochsson L, Gallagher AG, et al. Proficiency-based virtual reality training significantly reduces the error rate for residents during their first 10 laparoscopic cholecystectomies. Am J Surg. 2007;193(6):797-804.	No		VR training in cholecystectomy
102	Hoekema A et. al.	Hoekema A, Stegenga B, Bakker M, et al. Simulated driving in obstructive sleep apnoea-hypopnoea; effects of oral appliances and continuous positive airway pressure. Sleep Breath. 2007;11(3):129-138.	Does not simulate manual skills or surgery	1	Treatment modalities for OSAHS
103	Roy JD et. al.	Roy J-D, Massicotte L, Sassine M-P, Seal RF, Roy A. A comparison of intrathecal morphine/fentanyl and patient-controlled analgesia with patient-controlled analgesia alone for analgesia after liver resection. Anesth Analg. 2006;103(4):990-994.	Does not simulate manual skills or surgery	1	Pain management after liver ressection
104	Cohen J et. al.	Cohen J, Cohen SA, Vora KC, et al. Multicenter, randomized, controlled trial of virtual-reality simulator training in acquisition of competency in colonoscopy. Gastrointest Endosc. 2006;64(3):361-368.	No		Simulator training in colonoscopy
105	Anderson K et. al.	Anderson K, Gambhir S, Glavin R, Kinsella J. The use of an anaesthetic simulator to assess single-use laryngoscopy equipment. Int J Qual Health Care. 2006;18(1):17-22.	Only in simulation/no patients	1	Laryngoscopy and intubation
106	Chuang TY et. al.	Chuang T-Y, Sung W-H, Lin C-Y. Application of a virtual reality-enhanced exercise protocol in patients after coronary bypass. Arch Phys Med Rehabil. 2005;86(10):1929-1932.	Does not simulate manual skills or surgery	1	Exercise with VR after bypass surgery
107	Lorber MI et. al.	Lorber MI, Ponticelli C, Whelchel J, et al. Therapeutic drug monitoring for everolimus in kidney transplantation using 12-month exposure, efficacy, and safety data. Clin Transplant. 2005;19(2):145-152.	Does not simulate manual skills or surgery	1	Everolimus in patients after kidney transplantation
108	Starling RC et. al.	Starling RC, Hare JM, Hauptman P, et al. Therapeutic drug monitoring for everolimus in heart transplant recipients based on exposure-effect modeling. Am J Transplant. 2004;4(12):2126-2131.	Does not simulate manual skills or surgery	1	Everolimus in patients after heart transplantation
109	Edwards A et. al.	Edwards A, Elwyn G, Hood K, et al. Patient-based outcome results from a cluster randomized trial of shared decision making skill development and use of risk communication aids in general practice. Fam Pract. 2004;21(4):347-354.	Does not simulate manual skills or surgery	1	Effects of shared decision making
110	Steele E et. al.	Steele E, Grimmer K, Thomas B, Mulley B, Fulton I, Hoffman H. Virtual reality as a pediatric pain modulation technique: a case study. Cyberpsychol Behav. 2003;6(6):633-638.	Does not simulate manual skills or surgery	1	VR to modulate post-op pain
111	Schildhauer TA et. al.	Schildhauer TA, Ledoux WR, Chapman JR, Henley MB, Tencer AF, Routt MLC. Triangular osteosynthesis and iliosacral screw fixation for unstable sacral fractures: a cadaveric and biomechanical evaluation under cyclic loads. J Orthop Trauma. 2003;17(1):22-31.	Only in simulation/no patients	1	Modes of stabilizing pelvic fractures
112	Stratta RJ et. al.	Stratta RJ, Alloway RR, Hodge E, Lo A, Pancreas Investigators Vital Outcomes Trial (PIVOT) Study Group. A multicenter, open-label, comparative trial of two daclizumab dosing strategies vs. no antibody induction in combination with tacrolimus, mycophenolate mofetil, and steroids for the prevention of acute rejection in simultaneous kidney-pancreas transplant recipients: interim analysis. Clin Transplant. 2002;16(1):60-68.	Does not simulate manual skills or surgery	1	Regimens for preventing graft rejection after combined kidney and prancreas transplantation
113	Brown GC et. al.	Brown GC, Brown MM, Sharma S, Busbee B, Brown H. Incremental cost-effectiveness of laser therapy for choroidal neovascularization associated with histoplasmosis. Retina (Philadelphia, Pa). 2000;20(4):331-337.	Does not simulate manual skills or surgery	1	Cost effectiveness of laser photocoagulation therapy
114	Gilbart MK et. al.	Gilbart MK, Hutchison CR, Cusimano MD, Regehr G. A computer-based trauma simulator for teaching trauma management skills. Am J Surg. 2000;179(3):223-228.	Only in simulation/no patients	1	Management of trauma patients
115	Rogers PL et. al.	Rogers PL, Jacob H, Thomas EA, Harwell M, Willenkin RL, Pinsky MR. Medical students can learn the basic application, analytic, evaluative, and psychomotor skills of critical care medicine. Crit Care Med. 2000;28(2):550-554.	Only in simulation/no patients	1	Management of critically ill patients
116	Kylstra JA et. al.	Kylstra JA, Brown JC, Jaffe GJ, et al. The importance of fluorescein angiography in planning laser treatment of diabetic macular edema. Ophthalmology. 1999;106(11):2068-2073.	Does not simulate manual skills or surgery	1	Is flouresceuin angiography necessary before laser treatment of macular edema
117	Adamson J	Adamson J. Perisurgical use of epoetin alfa in orthopedic surgery patients. Semin Hematol. 1996;33(2 Suppl 2):55-58; discussion 59.	Does not simulate manual skills or surgery	1	EPO in patients before surgery
118	Gray DT et. al.	Gray DT, Thorburn J, Lundorff P, Strandell A, Lindblom B. A cost-effectiveness study of a randomised trial of laparoscopy versus laparotomy for ectopic pregnancy. Lancet. 1995;345(8958):1139-1143.	Does not simulate manual skills or surgery	1	Cost effectiveness of laparoscopy vs laparotomy in ectopic pregnancies
119	Barlow W et. al.	Barlow W, Azen S. The effect of therapeutic treatment crossovers on the power of clinical trials. The Silicone Study Group. Control Clin Trials. 1990;11(5):314-326.	Does not simulate manual skills or surgery	1	Treatment of retinal detachement
